# Evaluating the Impact of Assimilating Aerosol Optical Depth Observations on Dust Forecasts Over North Africa and the East Atlantic Using Different Data Assimilation Methods

**DOI:** 10.1029/2019MS001890

**Published:** 2020-04-04

**Authors:** Yonghan Choi, Shu‐Hua Chen, Chu‐Chun Huang, Kenneth Earl, Chih‐Ying Chen, Craig S. Schwartz, Toshihisa Matsui

**Affiliations:** ^1^ Department of Land, Air, and Water Resources University of California Davis CA USA; ^2^ Korea Polar Research Institute Incheon South Korea; ^3^ Research Center of Environmental Changes Academia Sinica Taipei Taiwan; ^4^ National Center for Atmospheric Research Boulder CO USA; ^5^ NASA Goddard Space Flight Center Greenbelt MD USA

**Keywords:** aerosol assimilation, dust model, GSI, aerosol optical depth (AOD), deep blue AOD, Sahara Desert

## Abstract

This study evaluates the impact of assimilating moderate resolution imaging spectroradiometer (MODIS) aerosol optical depth (AOD) data using different data assimilation (DA) methods on dust analyses and forecasts over North Africa and tropical North Atlantic. To do so, seven experiments are conducted using the Weather Research and Forecasting dust model and the Gridpoint Statistical Interpolation analysis system. Six of these experiments differ in whether or not AOD observations are assimilated and the DA method used, the latter of which includes the three‐dimensional variational (3D‐Var), ensemble square root filter (EnSRF), and hybrid methods. The seventh experiment, which allows us to assess the impact of assimilating deep blue AOD data, assimilates only dark target AOD data using the hybrid method. The assimilation of MODIS AOD data clearly improves AOD analyses and forecasts up to 48 hr in length. Results also show that assimilating deep blue data has a primarily positive effect on AOD analyses and forecasts over and downstream of the major North African source regions. Without assimilating deep blue data (assimilating dark target only), AOD assimilation only improves AOD forecasts for up to 30 hr. Of the three DA methods examined, the hybrid and EnSRF methods produce better AOD analyses and forecasts than the 3D‐Var method does. Despite the clear benefit of AOD assimilation for AOD analyses and forecasts, the lack of information regarding the vertical distribution of aerosols in AOD data means that AOD assimilation has very little positive effect on analyzed or forecasted vertical profiles of backscatter.

## Key Points

- Assimilating MODIS AOD data improves AOD forecasts up to 48 hr, but the improvement can last for only 30 hr when deep blue data are excluded
- The hybrid and EnSRF methods can produce better AOD analyses and forecasts than the 3D‐Var method does
- Assimilation of MODIS AOD data has very little impact on analyses and/or forecasts of aerosol backscatter profile

## 1 Introduction

Aerosols have received considerable attention globally due to their detrimental effects on air quality and public health (García‐Pando et al., 2014; Mallone et al., 2011; Morman & Plumlee, 2013; Pandolfi et al., 2014). Additionally, aerosols can influence socioeconomic activities (Prata & Tupper, 2009; Wilkinson et al., 2012), ecosystems (Jickells et al., 2005; Wang et al., 2015; Yu et al., 2015), the Earth's energy budget (Bond et al., 2013), and weather and climate (Boucher et al., 2013; Knippertz & Stuut, 2014; Lerach et al., 2008). Although important research on aerosols has advanced our understanding of their characteristics and impacts on weather and climate, a great deal of uncertainty still exists in aerosol modeling due to an incomplete understanding of emission processes (Astitha et al., 2012; Spada et al., 2013; Textor et al., 2007), aerosol‐radiation‐cloud interactions (Kinne et al., 2006; Mann et al., 2014; Wang et al., 2014), and aerosol aging processes (Abdelkader et al., 2017; Lee et al., 2016; Lund et al., 2017; Textor et al., 2006), as well as errors in initial and boundary conditions. This study contributes to efforts that reduce aerosol forecast uncertainty through the assimilation of aerosol observations.

Aerosol observations (e.g., aerosol optical depth [AOD]) have been assimilated, along with meteorological observations (e.g., temperature and wind) from ground‐based, airborne, and spaceborne instruments, to improve the initial conditions for aerosol forecasts using a variety of data assimilation (DA) techniques, including the three‐dimensional variational (3D‐Var) method (D. Chen et al., 2017; Jiang et al., 2013; Li et al., 2013; Saide et al., 2013), ensemble‐based method (Di Tomaso et al., 2017; Pagowski & Grell, 2012; Peng et al., 2017; Rubin et al., 2016, 2017; Schutgens et al., 2010a, 2010b; Sekiyama et al., 2010), and hybrid method (Lee et al., 2017; Schwartz et al., 2014). For example, Liu et al. (2011) implemented 3D‐Var assimilation for AOD observations into the National Centers for Environmental Prediction (NCEP) Gridpoint Statistical Interpolation (GSI) 3D‐Var DA system and used it to assimilate moderate resolution imaging spectroradiometer (MODIS) AOD data from the Terra and Aqua satellites for a dust storm case over East Asia. They showed that both aerosol analyses and subsequent aerosol forecasts were improved by assimilating AOD observations. Building upon Liu et al. (2011), Schwartz et al. (2012) assimilated MODIS AOD and AIRNow surface observations of particulates with radii ≤2.5 μm (PM_2.5_) using the NCEP GSI 3D‐Var DA system. Both AOD and surface PM_2.5_ forecasts over the United States showed the greatest improvement when AOD and PM_2.5_ observations were assimilated concurrently. Recently, Benedetti et al. (2019) assimilated MODIS AOD retrievals from both dark target and deep blue algorithms using the European Centre for Medium‐Range Weather Forecasts (ECMWF) operational 4D‐Var system and showed that the assimilation of MODIS AOD observations, especially deep blue products, could improve dust analyses and forecasts over the East Asia up to 48 hr.

Although the 3D‐Var technique can improve aerosol analyses and forecasts, the use of static (i.e., time‐invariant) background error covariances (BECs) limits its usefulness in some situations. Conversely, ensemble‐based DA techniques, such as the ensemble Kalman filter (EnKF; Evensen, 1994; Houtekamer & Mitchell, 1998), ensemble square root filter (EnSRF; Whitaker & Hamill, 2002), and ensemble adjustment Kalman filter (EAKF; Anderson, 2001), can spread observation information beyond the observation locations more effectively than variational techniques through flow‐dependent BECs. However, ensemble‐based methods can suffer from sampling errors due to a limited number of ensemble members, but these can be mitigated through the optimal use of covariance localization and inflation. Pagowski and Grell (2012) assimilated surface PM_2.5_ observations from the United States Environmental Protection Agency AIRNow network using the NCEP GSI 3D‐Var and EnSRF DA systems. Their results demonstrated the superiority of the EnSRF method over the 3D‐Var method in forecasting aerosol concentrations. In contrast, Rubin et al. (2016) found that the differences between aerosol analyses produced by assimilating MODIS AOD using an EAKF from the National Center for Atmospheric Research (NCAR)'s Data Assimilation Research Testbed and a 2D‐Var method from the Naval Research Laboratory Atmospheric Variational Data Assimilation System (NAVDAS) were statistically insignificant in terms of root mean square (RMS) error (RMSE), bias, and correlation. Similar to Rubin et al. (2016), Schwartz et al. (2014) found that the aerosol analyses and forecasts produced by the EnSRF failed to outperform those produced by the 3D‐Var method due to insufficient ensemble spread.

Recently, ensemble‐variational hybrid DA techniques have become increasingly popular for meteorological applications and are used in operational analysis systems at the United Kingdom Meteorological Office (Clayton et al., 2013), NCEP (Kleist & Ide, 2015a, 2015b), and Environment Canada (Buehner et al., 2015; Caron et al., 2015). Hybrid techniques enjoy the benefits of ensemble‐based flow‐dependent BECs while also using static BECs to mitigate the potential sampling issues within ensemble‐based methods. Schwartz et al. (2014) assimilated MODIS AOD, surface PM_2.5_, and conventional meteorological observations over the United States using 3D‐Var, EnSRF, and hybrid variational‐ensemble methods within the NCEP GSI DA and found that aerosol forecasts initialized from hybrid analyses best matched AIRNow and Aerosol Robotic Network (AERONET) observations in terms of bias, RMSE, correlation, and Equitable Threat Score.

Following the approach of Schwartz et al. (2014), this study assesses the impact of assimilating AOD and other meteorological observations on dust forecasts over North Africa and the East Atlantic using the 3D‐Var, EnSRF, and hybrid DA methods within the GSI system and the Weather Research and Forecasting (WRF) dust model (S.‐H. Chen et al., 2010, 2015). However, this study differs from Schwartz et al. (2014) in three main aspects. First, Schwartz et al. (2014) evaluated the effects of assimilating aerosol observations on aerosol forecasts over the United States, while this study focuses on dust forecasts over North Africa and the East Atlantic during the summer, where dust transport is dominated by strong flow features (e.g., African easterly jet and African easterly waves). In addition, to the best of the authors' knowledge, this is the first study to examine the impact of hybrid method aerosol DA on dust forecasts over the Sahara Desert, which is the largest contributor to global mineral dust aerosol emissions (Prospero et al., 2002). Second, Schwartz et al. (2014) assimilated AOD data produced by MODIS's “dark target” algorithm, which is used to retrieve AOD values over dark land, such as forest (Kaufman et al., 1997; Levy et al., 2013, 2015) and “ocean retrieval” algorithm (Remer et al., 2005; Tanré et al., 1999), while this study assimilates AOD from the dark target, ocean retrieval, and deep blue algorithms (Sayer et al., 2013). The deep blue algorithm, which is used to retrieve AOD data over reflective land surfaces (e.g., desert and barren land), provides a vast amount of additional AOD data over North African deserts. Analyses and forecasts of dust over North Africa and the East Atlantic, which primarily originates from North African deserts, could benefit immensely from high‐quality deep blue AOD data over those desert regions. Substantial improvements to the deep blue algorithm have increased the viability of deep blue AOD data for DA applications, particularly in MODIS data collection 6.1 (Korras‐Carraca et al., 2015; Sayer et al., 2013, 2014), easing the data quality concerns that have often resulted in deep blue AOD being excluded from AOD DA studies (e.g., Schwartz et al., 2012, 2014). Third, in addition to AOD data, Schwartz et al. (2014) assimilated solely conventional meteorological observations while this study assimilates both conventional and satellite radiance observations. The exclusion of satellite data in Schwartz et al. (2014) might have caused them to overestimate the impact of AOD assimilation on aerosol forecasts.

The remainder of this work is organized as follows: A brief description of the numerical tools used in this study is given in section 2. In section 3, the observations used and numerical experiment design are described. Dust aerosol analyses and forecasts from different experiments are compared and discussed in section 4, and a concluding summary is presented in section 5.

## 2 Dust Model, Data Assimilation System, and Their Configurations

### 2.1 Dust Model

A dust model, based on the WRF model Version 3.7.1 (Skamarock et al., 2008), was developed by S.‐H. Chen et al. (2010, 2015) and is used for dust and weather forecasts. Only a brief introduction of the dust model is given here as a thorough description of the model can be found in the original papers. A dust continuity equation in the model accounts for transport, subgrid boundary layer mixing, subgrid cumulus mixing, sedimentation, and other source/sink processes. In this study, five size bins with median dust radii of 0.25, 0.5, 1.0, 2.0, and 4.0 μm are used. These size bins are also used in the assimilation system, which is introduced in section 2.2.

Dust is introduced to the model via surface emission and is removed via dry deposition to the ground, wet scavenging by cloud droplets, and rainfall. Emission is controlled by three parameters, as described in S.‐H. Chen et al. (2010)—vegetation type, soil moisture, and 10‐m wind speed—and it is active when (1) the vegetation type is “barren,” (2) soil volumetric moisture content is less than 0.2, and (3) 10‐m wind speed is greater than a threshold value, which is set to 6.0 m s^−1^ in this study.

In the dust model, the National Aeronautics and Space Administration (NASA)/Goddard Space Flight Center shortwave and longwave radiation schemes (Chou et al., 2001; Chou & Suarez, 1994, 1999) were modified to consider dust‐radiation interaction. Dust aerosol optical properties (i.e., single scattering albedo, asymmetry parameter, and extinction coefficient) with respect to different wavelengths were calculated using the Optical Properties of Aerosols and Cloud software package (Hess et al., 1998). Dust‐microphysics interaction was also included in the two‐moment microphysics scheme (Cheng et al., 2010) that was implemented in the dust model. Dust particles can serve as both cloud condensation nuclei and ice nuclei. For the ice nuclei, two heterogeneous ice nucleation processes are included: deposition and immersion freezing (J.‐P. Chen et al., 2008; Hoose et al., 2010).

### 2.2 Data Assimilation System

The GSI (Shao et al., 2016) is an analysis system that was originally developed for operational use but is now freely available for community use. The system is designed to be flexible and can be run efficiently on various parallel computing platforms. Three different DA methods are available in the GSI analysis system, including the 3D‐Var method, EnSRF method, and hybrid method, in which the last one combines the first two. Below, we briefly introduce how AOD observations are assimilated in each method.

#### 2.2.1 The 3D‐Var Method

Liu et al. (2011) extended the Community Radiative Transfer Model (Han et al., 2006; Liu & Weng, 2006) to implement an AOD observation operator into the GSI analysis system. The forward operator computes AOD using aerosol profiles as an input. Liu et al. (2011) employed 3D mass concentrations of aerosol species as control variables (“one‐step procedure”). This is different from previous studies (e.g., Benedetti et al., 2009; Lee et al., 2013; Pagowski et al., 2010; Zhang et al., 2008) that employed either total column AOD or total aerosol mass as control variables (“two‐step procedure”), requiring assumptions of the vertical distribution and contribution of individual species' mass to the total aerosol mass. The control variables of the 3D‐Var method include unbalanced surface pressure, stream function, unbalanced velocity potential, unbalanced virtual temperature, and pseudo relative humidity. In addition, the control variables also include the mixing ratios of five bin dust variables. The code that was developed by Liu et al. (2011) is modified and used in our 3D‐Var AOD assimilation. Note that in Liu et al. (2011), there are 15 aerosol species, including five different dust species. Since dust is the major aerosol type over the region of interest and is the only aerosol type in the WRF dust model, we set other 10 aerosol species to zeros so that the assimilation of AOD only corrects five dust species.

The BEC was calculated using the National Meteorological Center method (Parrish & Derber, 1992). In detail, background error statistics were computed using the differences between 24‐ and 12‐hr forecasts initialized at 0000 or 1200 UTC each day during the month of June 2015. Cross correlations between different aerosol variables or between aerosol and meteorological variables are not considered, and only some multivariate correlations between stream function and velocity potential/virtual temperature/surface pressure are taken into account.

#### 2.2.2 The EnSRF Method

The community EnKF system released with the NCEP GSI system is based on the EnSRF, and model‐simulated observations are computed using the GSI observation operators, including AOD, as those used for 3D‐Var. In the EnSRF, aerosol control (or analysis) variables are identical to those in 3D‐Var, and meteorological control variables are zonal wind, meridional wind, temperature, pseudo relative humidity, and surface pressure. In this study, cross correlations are not permitted between meteorological and aerosol analysis variables. However, cross correlations between the five meteorological analysis variables are allowed as are cross correlations between the five size bin dust variables.

In this study, 36 ensemble members were used to calculate flow‐dependent BECs. Both inflation and covariance localization are used to alleviate the sampling problem caused by the use of a limited number of ensemble members. Posterior multiplicative “relaxation to prior spread” inflation (Whitaker & Hamill, 2012) was adopted to maintain ensemble spread and avoid filter divergence. The inflation factors for meteorological and aerosol variables were set to 1.12 and 1.20, respectively, following Schwartz et al. (2014). The horizontal and vertical covariance localizations, which were used to reduce spurious correlations, were based upon the Gaspari and Cohn function (Gaspari & Cohn, 1999). The horizontal localization scale for all observations (i.e., conventional, surface pressure, radiance, and AOD observations) is 1,800 km following Schwartz et al. (2014), and vertical localization scales for conventional/AOD and radiance/surface pressure observations are 1.0 and 2.2 scale height, respectively, following Wang et al. (2013). Note that the inflation method and horizontal/vertical localization scales used are determined from the information contained in previous studies as well as an analysis of our own sensitivity tests (results not shown). Observation‐space vertical localization used in EnSRF requires a vertical location for each observation. Because AOD observations are vertically integrated quantities, to assign vertical locations for AOD observations is not straightforward. Therefore, the model‐simulated aerosol transmittance for each AOD observation was calculated, and the pressure level where the transmittance was maximized was assigned as the vertical location for each AOD observation. Note that this approach is similar to the widely used method for determining the vertical locations for the assimilation of satellite radiances (Hamill et al., 2011; Houtekamer & Mitchell, 2005; Houtekamer et al., 2005; Newman et al., 2015).

#### 2.2.3 The Hybrid Method

In the NCEP GSI hybrid analysis system, a combination of static BEC, which is used for 3D‐Var, and flow‐dependent BEC, which is used for EnSRF, is employed through the use of extended control variables (Lorenc, 2003). The specifics of expansion of the GSI hybrid system for aerosol DA are given in Schwartz et al. (2014), and only important aspects of aerosol DA are briefly described here.

As mentioned in Schwartz et al. (2014), the number of extended control variables is reduced in the GSI hybrid system for computational efficiency. This approach introduces cross correlations between all control variables, including cross correlations between meteorological and aerosol variables. Auxiliary experiments are conducted for a limited number of cases to investigate effects of the meteorological‐aerosol correlations included in the BEC of the hybrid method. In the auxiliary experiments, meteorology and aerosol observations are assimilated using the EnSRF method that includes cross correlations between meteorological and aerosol variables. Results of the auxiliary experiments are qualitatively similar to results with the EnSRF method without the meteorological‐aerosol correlations, which is consistent with section 7 of Schwartz et al. (2014). In this study, in combining static and flow‐dependent BECs, the weights given to the static and flow‐dependent BECs are 0.25 and 0.75, respectively. Limited sensitivity to the weights given to BECs from 3D‐Var and EnKF was reported for meteorological (Wang, 2011; Wang et al., 2013; Zhang et al., 2013) and aerosol applications (Schwartz et al., 2014). Several values for the weights given to the static and flow‐dependent BECs are tested, but, as in previous studies, the resulting analyses show little sensitivity to the weighting values chosen. Covariance localization was also used in the hybrid to reduce spurious correlations, with identical values as in the EnSRF. Note that vertical localization in hybrid is model‐space localization (cf. observation‐space localization in EnSRF) and does not require an exact vertical location of each observation.

## 3 Observations, Ensembles, and Experimental Design

### 3.1 Observations for Assimilation

Observations that are used in DA include AOD, satellite radiance, and conventional data. DA is cycled every 6 hr with observations falling within a 6‐hr time period centered at the analysis time. AOD observations are provided by MODIS Level 2 (L2) 550‐nm AOD retrievals from the Terra and Aqua satellites, which are gridded at a 10‐km horizontal resolution. Unlike Schwartz et al. (2014), who only assimilated dark target AOD data, this study uses both dark target and deep blue data. Only observations of AOD < 5.0 with the “best” quality, as determined by a confidence flag value of 3, are considered for assimilation. All AOD observations meeting these criteria are thinned to a 54‐km grid to reduce correlations between adjacent observations.

**Figure 1:**
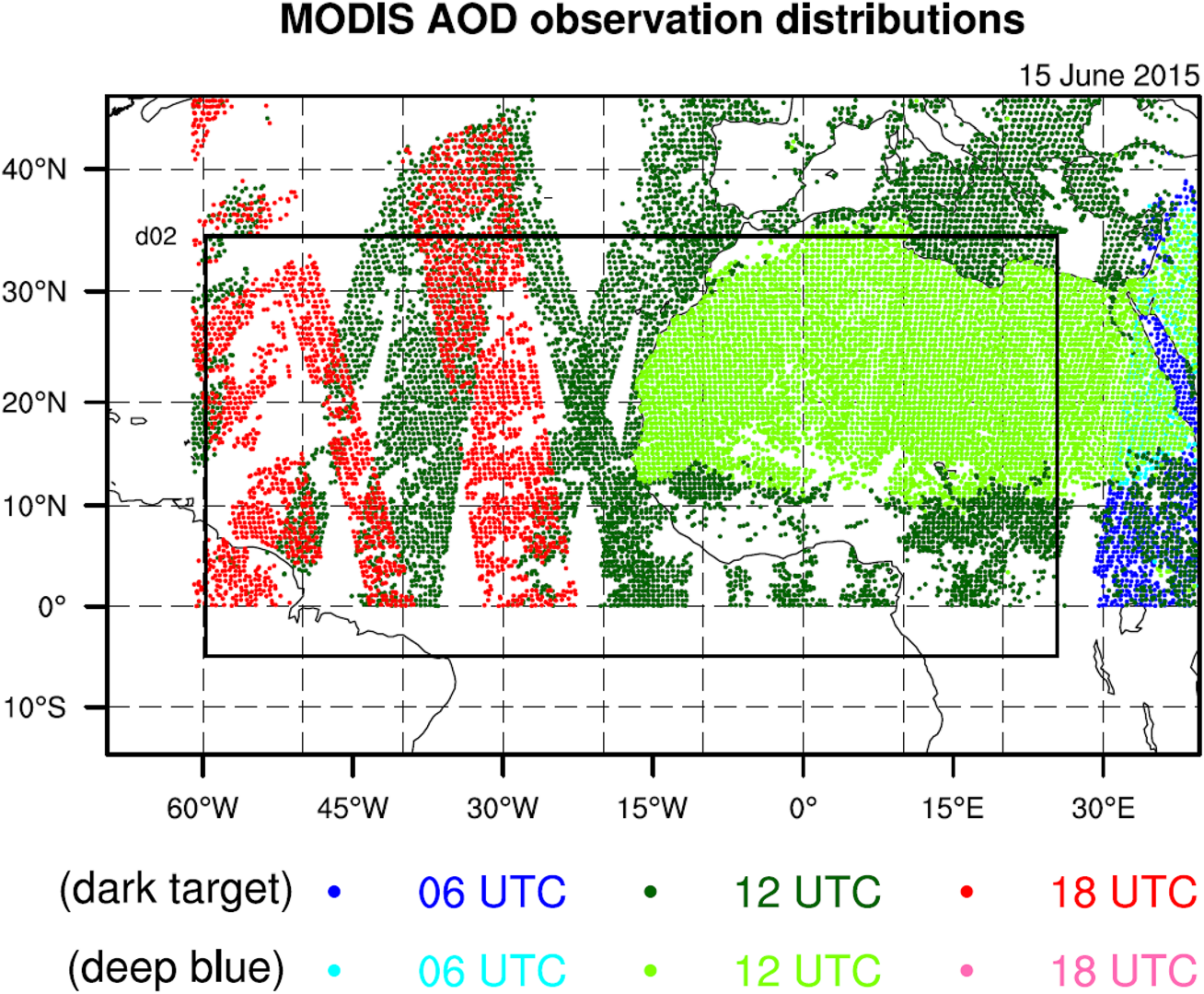
MODIS AOD observations (both deep blue and dark target) assimilated at 0600 (blue), 1200 (green), and 1800 (red) UTC 15 June 2015 over Domain 1. Only observations north of the equator and east of 60°W are assimilated. Boundaries of Domain 2 are also shown.

**Figure 2:**
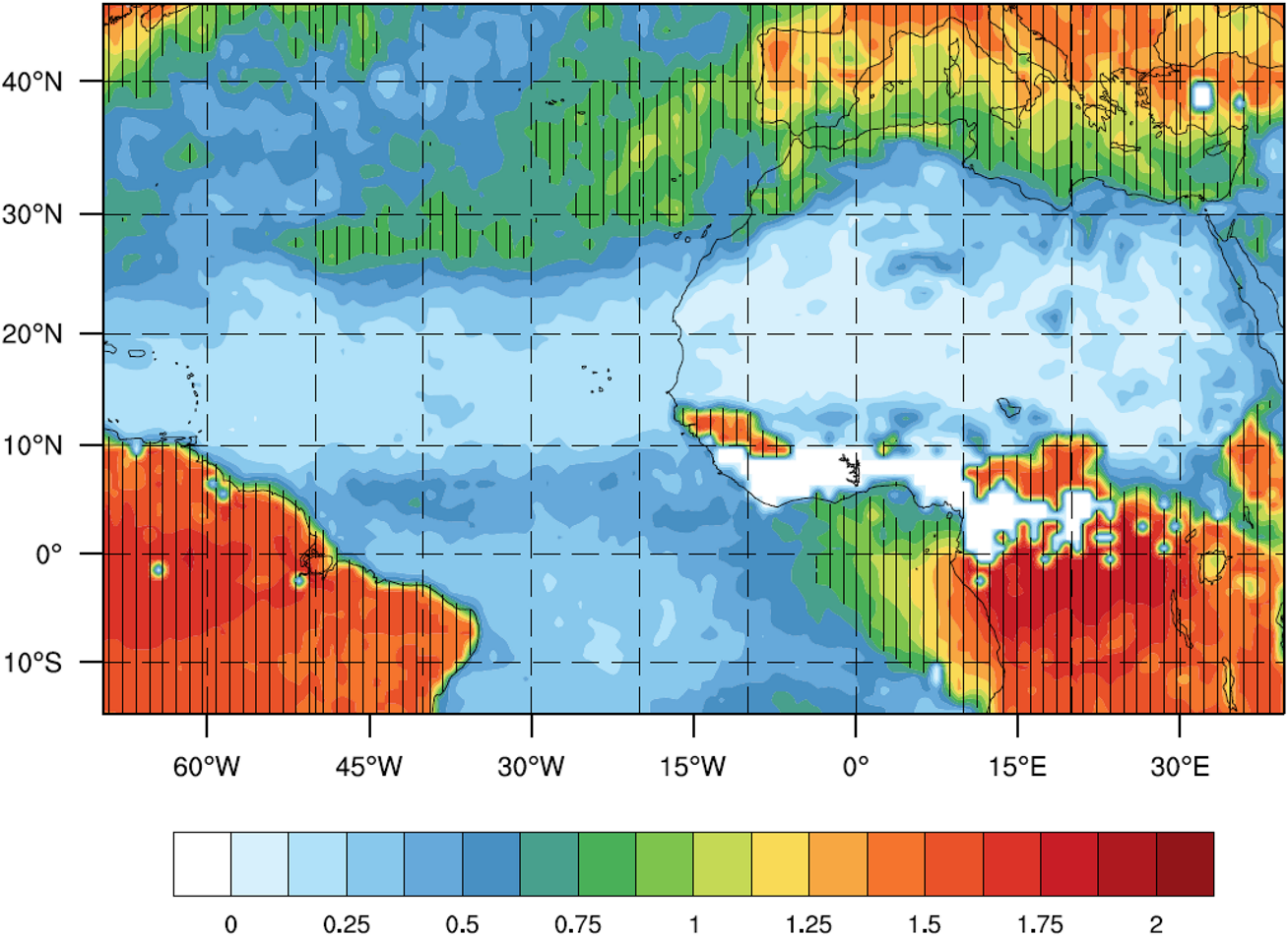
Monthly averaged (June 2015) Ångström exponent (AE) from the Visible Infrared Imaging Radiometer Suite (VIIRS) sensor. Hatched regions indicate AE values greater than 0.75.

MODIS AOD data are only available during the day. Thus, AOD observations can only be assimilated at 0600, 1200, and 1800 UTC for our model domain, which includes North Africa and the East Atlantic. Figure 1 gives an example of typical distributions of MODIS AOD observations (both deep blue and dark target products) at 0600, 1200, and 1800 UTC. Since only dust aerosols are considered in this study, we have attempted to reduce the amount of nondust aerosols that, through AOD DA, are ultimately treated as dust in the WRF dust model by only assimilating AOD observations north of the equator and east of 60°W, which prevents the assimilation of most AOD observations dominated by biomass burning or sea salt aerosols. To confirm that the assimilated AOD observations are mainly due to the presence of dust aerosols, monthly averaged Ångström exponent (AE) values from the Visible Infrared Imaging Radiometer Suite (VIIRS) sensor are plotted (Figure 2). AE values can be used as an indicator of aerosol size, with smaller values generally corresponding to larger aerosols, such as dust. The VIIRS AE values are calculated based on the 488‐ and 670‐nm optical depths over land and the 550‐ and 865‐nm optical depths over the ocean. Although some MODIS AOD observations north of the equator and east of 60°W (e.g., north of North Africa and north of 25°N over the Atlantic) correspond to AE values greater than 0.75 (a threshold value used in Di Tomaso et al., 2017), most assimilated MODIS AOD observations have AE values smaller than 0.75. We will use the filtering method in Di Tomaso et al. (2017) to exclude nondust AOD observations in our future work.

The observational error of MODIS AOD data depends on the retrieval algorithm (i.e., dark target versus deep blue) and the surface type (water, nondesert land, or desert). Errors for dark target data are assigned following the method used by Remer et al. (2005), Schwartz et al. (2012), and Schwartz et al. (2014). Although the dark target observational errors used in this study are smaller than those referenced in previous studies (e.g., Hyer et al., 2011; Shi et al., 2011; Zhang & Reid, 2006), they are consistent with values that were successfully used in previous studies (Liu et al., 2011; Schwartz et al., 2012; Schwartz et al., 2014). For deep blue data, observational errors are determined using each observation's uncertainty information and confidence flag value, which are included in L2 MODIS AOD/aerosol data files. The actual formulae used to compute observational errors for any MODIS AOD (*ε*_AOD_) observation are as follows:

$$\varepsilon_{\mathrm{AOD}}=\left\{\begin{array}{l} 0.03+0.05\tau_{o},\;\text{dark target}\;\left(\text{over water}\right)\\ 0.05+0.15\tau_{o},\;\text{dark target}\;\left(\text{over land}\right)\;\\ 0.05+0.15\tau_{o}+0.01\left(3-\text{dbcf}\right),\;\text{deep blue}\; \end{array}\right.$$

where *τ*_o_ is the MODIS AOD value and *dbcf* is the confidence flag for deep blue products.

The satellite radiance observations assimilated in this study are listed in Table 1. Channel selection for each instrument and observational error for each channel are consistent with NCEP's operational settings. Prior to assimilation, all radiance observations are thinned to a 108‐km grid and quality screened within the GSI system. Details of GSI's quality control (QC) procedures for radiance data can be found in Hu et al. (2016). Since radiance observations are prone to systematic errors (i.e., biases), we use GSI's variational bias correction (VarBC) capability (Auligné et al., 2007; Dee, 2004; Derber & Wu, 1998; Pu et al., 2019; Zhu, Derber, Collard, Dee, Treadon, Gayno, & Jung, 2014; Zhu, Derber, Collard, Dee, Treadon, Gayno, Jung, Groff, et al., 2014) to limit their impact on assimilation. Initial bias correction coefficients for our specific assimilation settings, observation data, model configuration, and time period are generated following the method described by Hu et al. (2016) and Liu et al. (2012) and updated during each DA cycle.

**Table 1:** Satellite Radiance Observations Assimilated

Instrument		Satellite
Infrared	High‐Resolution Infrared Radiation Sounder‐3 (HIRS‐3)	NOAA‐16, 17
	HIRS‐4	MetOp‐A, B NOAA‐18, 19
	Atmospheric Infrared Sounder (AIRS)	Aqua
	Infrared Atmospheric Sounding Interferometer (IASI)	MetOp‐A, B
	Cross‐track Infrared Sounder (CrIS)	Suomi NPP
Microwave	Advanced Microwave Sounding Unit‐A (AMSU‐A)	NOAA‐15, 18, 19 MetOp‐A, B Aqua
	AMSU‐B	NOAA‐17
	Microwave Humidity Sounder (MHS)	NOAA‐18, 19 MetOp‐A, B
	Advanced Technology Microwave Sounder (ATMS)	Suomi NPP
	Advanced Microwave Scanning Radiometer for EOS (AMSR‐E)	Aqua
	Special Sensor Microwave Imager (SSM/I)	DMSP F‐13, 14, 15
	Special Sensor Microwave Imager/Sounder (SSMI/S)	DMSP F‐16

Finally, we assimilate conventional, Global Positioning System (GPS) Radio Occultation (RO), and satellite‐derived wind observations, which are listed in Table 2. These data are also subject to a battery of QC checks within GSI before assimilation. Observational errors for conventional observations follow those from the North American Mesoscale Forecast System.

**Table 2:** Conventional Observations Assimilated

Observing platform	Observation type
Radiosonde	Wind temperature Specific humidity Surface pressure
Aircraft	Wind temperature
Wind profiler	Wind
Ship and buoy	Wind temperature Specific humidity Surface pressure
Surface Synoptic Observation (SYNOP) and Meteorological Aerodrome Reports (METAR)	Surface pressure
GPS RO	Refractivity
Satellite‐derived winds	Wind

### 3.2 Configuration of the Ensemble

Thirty‐six ensemble members are used in EnSRF DA. The horizontal grid spacing of each ensemble member is 27 km. Following Torn et al. (2006), the initial and lateral boundary conditions (at 0000 UTC 27 May 2015) for the ensemble members are generated by perturbing the control variables, including dust aerosols, from the values derived from NCEP's Final (FNL) global analysis. Perturbations are randomly assigned according to a Gaussian probability distribution with a zero mean and standard deviation equal to the static BEC used in 3D‐Var.

Although we are primarily interested in analyses and forecasts produced for June 2015, the ensemble starts DA cycling 5 days earlier at 0000 UTC 27 May 2015 to improve the ensemble spread from the random perturbations for dust and other meteorological variables. The 5‐day DA cycling assimilates both meteorological and AOD observations mentioned in section 3.1 using the EnSRF DA method. The last forecast ensemble of the 5‐day DA cycling, which is initialized by the analysis ensemble at 1800 UTC 31 May, will be used for 1‐month DA cycling experiments in this study, and it is referred to the spun‐up forecast ensemble in the remainder of this paper.

### 3.3 Experimental Design

A total of six primary DA cycling experiments is conducted (Table 3). An additional experiment (Hybrid_NoDB), which is introduced in section 4.3, is performed to examine the impact of assimilating MODIS deep blue AOD observations. All experiments assimilate conventional, GPS RO, satellite‐derived winds, and radiance observations. The first part of any experiment name (i.e., “3DVAR,” “EnKF,” or “Hybrid”) in Table 3 indicates the DA method used for the experiment, while the second part of the experiment name indicates whether AOD observations (both dark target and deep blue data) are (“ALL”) or are not (“NoAOD”) assimilated. Comparing the results of experiments that differ only in the DA method used allows one to assess the relative performance of each DA method, while comparing experiments that differ only by whether or not they assimilate AOD observation permits one to investigate the impact of AOD assimilation on dust forecasts.

**Table 3:** Summary of DA Cycling Experiments

Experiment name	DA method	Assimilated observations
3DVAR_ALL	3D‐Var	AOD (deep blue and dark target), conventional, and radiance observations
EnKF_ALL	EnSRF	
Hybrid_ALL	Hybrid	
3DVAR_NoAOD	3D‐Var	Conventional and radiance observations
EnKF_NoAOD	EnSRF	
Hybrid_NoAOD	Hybrid	
Hybrid_NoDB	Hybrid	AOD (dark target only), conventional, and radiance observations

All experiments conduct analysis‐forecast cycles every 6 hr from 0000 UTC 1 June 2015 to 1800 UTC 30 June 2015. For the first cycle, the first‐guess or background information for all experiments is derived from the spun‐up forecast ensemble valid at 0000 UTC 1 June 2015, with the “EnKF” experiments using the full ensemble and the “3DVAR” and “Hybrid” experiments using the mean of the ensemble. Background information for each subsequent cycle is taken from, in the case of the “EnKF” experiments, a 6‐hr dust model forecast ensemble initialized from the most recent posterior (analysis) ensemble or, in the case of the “3DVAR” or “Hybrid” experiment, a deterministic 6‐hr dust model forecast initialized from the most recent analysis. It should be noted that posterior ensembles were not recentered about hybrid analyses (i.e., there is no feedback from hybrid cycle to EnSRF cycle).

The posterior ensemble means of the “EnKF” experiments and analyses of the “3DVAR” and “Hybrid” experiments for all cycles (i.e., from 0000 UTC 1 to 1800 UTC 30 June 2015 at 6‐hr intervals) are used to initialize 72‐hr forecasts, producing a total of 120 forecasts for each experiment. Each 72‐hr dust model forecast consists of two nested domains with horizontal grid spacings of 27 and 9 km, respectively (Figure 1), 38 vertical levels, and a 50‐hPa model top. All forecasts are made with the two‐way nesting option and use the physics schemes listed in Table 4. Note that only the outer 27‐km domain is used in the DA cycling process.

**Table 4:** Physics Schemes Used for Dust Forecasts

Physical parameterization	Selected option
Microphysics	Two‐moment scheme (Cheng et al., 2010)
Cumulus parameterization	Kain‐Fritsch scheme (Kain, 2004)
Planetary boundary layer	MRF scheme (Hong & Pan, 1996)
Shortwave radiation	GSFC scheme (Chou & Suarez, 1999)
Longwave radiation	GSFC scheme (Chou et al., 2001)
Land surface model	Noah LSM (F. Chen et al., 1996)

## 4 Results and Discussions

### 4.1 DA Analysis

The analyses from all 6‐hourly DA cycles are, unless otherwise specified, evaluated against three different observational data sets, including MODIS and AERONET AODs and aerosol backscatter profiles from the Cloud‐Aerosol Lidar with Orthogonal Polarization (CALIOP) instrument aboard the Cloud‐Aerosol Lidar and Infrared Pathfinder Satellite Observation (CALIPSO) satellite. All comparisons are performed at the observation locations (i.e., analyses are interpolated to observation locations). RMSEs and biases are used to evaluate the performance of each DA method.

#### 4.1.1 Comparison With MODIS AOD Observations

**Figure 3:**
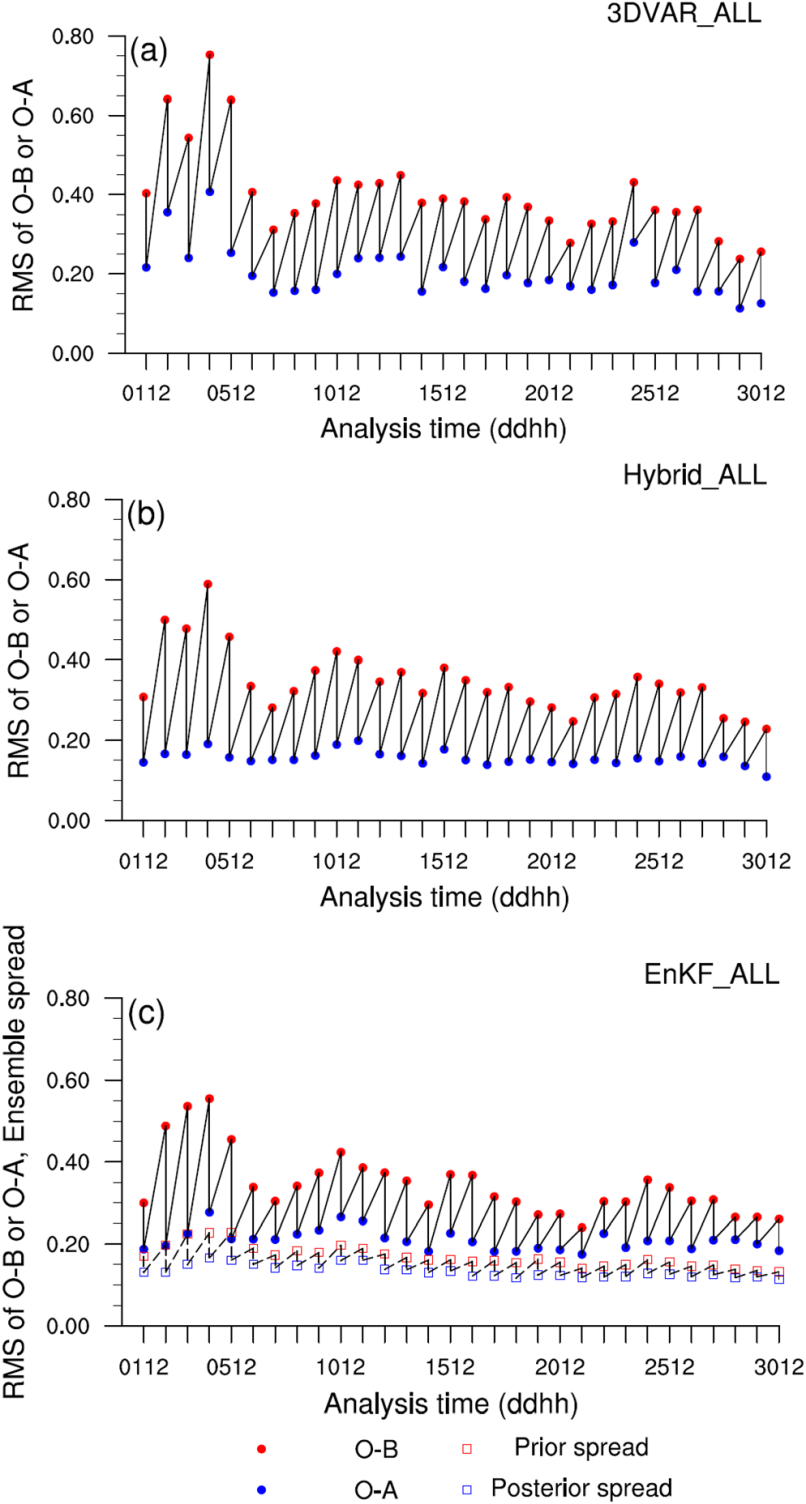
Root mean square (RMS) of O − B (red circle) and O − A (blue circle) for 550‐nm MODIS AOD, computed using all 1200 UTC analyses of (a) 3DVAR_ALL, (b) Hybrid_ALL, and (c) EnKF_ALL experiments. For EnKF_ALL experiment, prior (red square) and posterior (blue square) total spreads are also shown. The numbers on abscissa denote day and hour in June 2015.

The results from the three “ALL” experiments, all of which assimilate MODIS AOD observations, are used to confirm that AOD assimilation is proceeding as expected. Figure 3 shows RMS of O − B (observation minus background) and O − A (observation minus analysis) for AOD for all 1200 UTC DA cycles. The 1200 UTC cycle is used since the amount of MODIS data available over North Africa and the East Atlantic is maximized during the 1200 UTC cycle's DA time window. The RMS of O − A is smaller than the RMS of O − B for all analysis cycles, and the difference between the two RMS values decreases with each additional DA cycle as expected.

For an ensemble‐based DA system, the spread‐skill relationship is also often used to examine whether the system is stable and performs reasonably. The total spread is defined as the square root of the sum of the observation error variance and ensemble variance of the simulated observations. A system is considered well calibrated if the total spread of the prior ensemble is similar to the magnitude of the RMS difference (RMSD) between the prior ensemble mean B and the observations O (Houtekamer et al., 2005; Schwartz et al., 2014). For the EnKF_ALL experiment, the total spread for both the prior and posterior ensembles remains nearly constant throughout the cycling period, indicating that the EnSRF system is stable. However, the prior ensemble's total spread is smaller than RMS of O − B for all cycles, indicating that the performance of the EnSRF system is suboptimal in terms of its spread‐skill diagnostics. Note that the results of the 0600 and 1800 UTC DA cycles produce the same conclusions as those of the 1200 UTC DA cycle.

**Figure 4:**
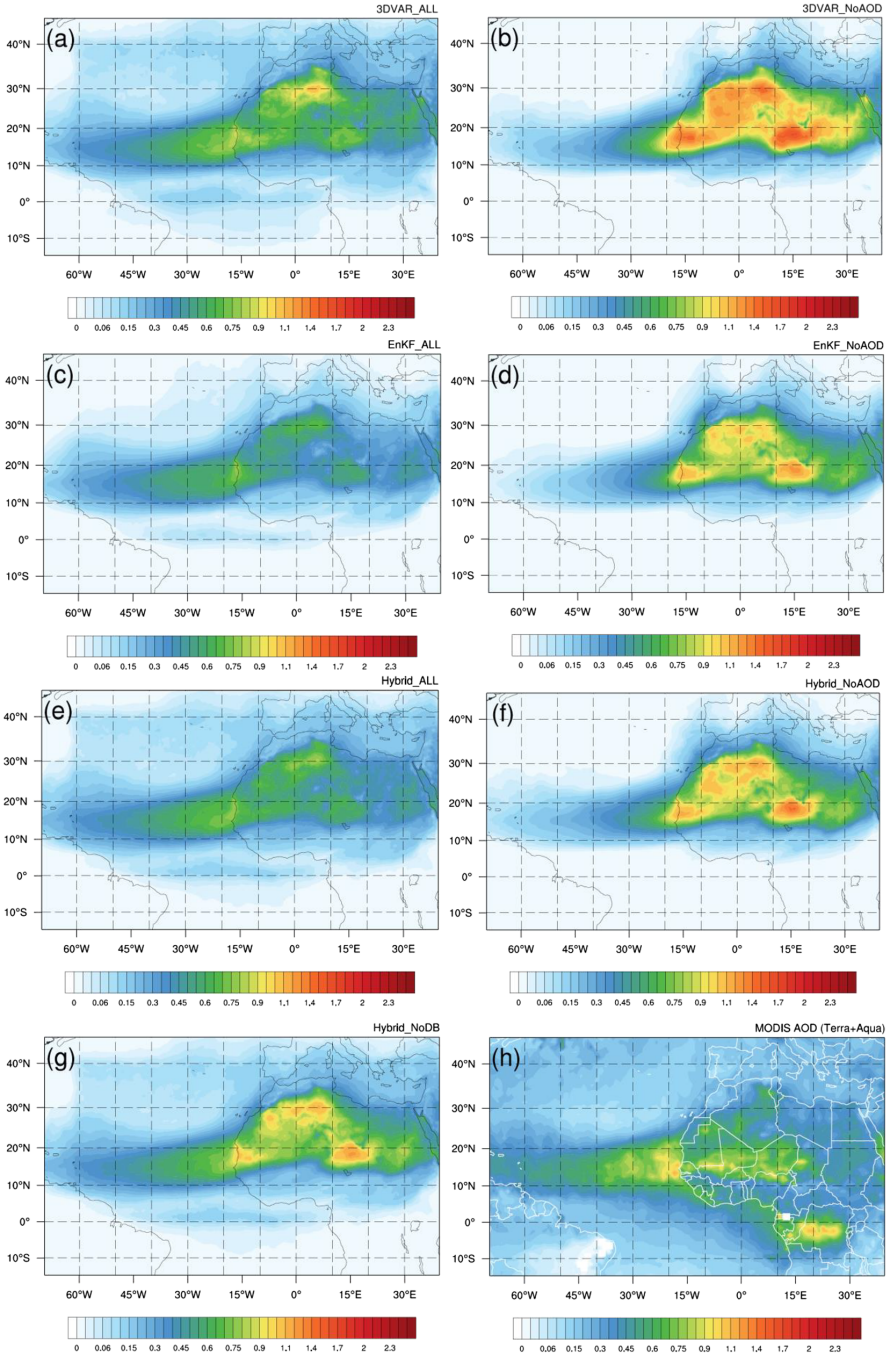
Monthly averaged AOD distribution of June 2015 for (a) 3DVAR_ALL, (b) 3DVAR_NoAOD, (c) EnKF_ALL, (d) EnKF_NoAOD, (e) Hybrid_ALL, (f) Hybrid_NoAOD, (g) Hybrid_NoDB analyses, and (h) MODIS AOD observation.

Figure 4h shows the MODIS monthly‐average 550‐nm AOD distribution for June 2015, produced by merging data from the Terra and Aqua satellites. The monthly‐average MODIS AOD field is the average of the MODIS AOD data fields produced at each analysis time by interpolating MODIS AOD observations (from both the Terra and Aqua satellites) available within a 6‐hr window—centered on the analysis time—to the model grid. Interpolation is accomplished by computing the distance‐weighted average of all observations present within 30 km of each grid point. Large AOD values over Mauritania, Western Sahara, Mali, Algeria, Niger, and Chad are associated with mineral dust originating from the Sahara Desert. High AOD values over the East Atlantic are primarily associated with the transport of Saharan dust by the prevailing wind (e.g., African Easterly Jet). The AOD “discontinuity” appearing along the western coast of North Africa is due to the use of different retrieval algorithms for bright desert land surfaces (deep blue) and ocean surfaces (ocean retrieval). Significant AOD features over Gabon, the Republic of the Congo, and the Democratic Republic of the Congo are primarily due to biomass burning in the Congo Basin, which produces black and organic carbon. Small but nonnegligible AOD values over the West Atlantic can be primarily attributed to sea salt aerosols ejected from the ocean surface via bubble‐bursting processes. Since sea salt, organic carbon, and black carbon aerosols are not considered in this study (i.e., only dust aerosol is included in the WRF dust model), special caution should be exercised when interpreting and evaluating the AOD analyses for areas where dust is not the dominant aerosol type.

**Figure 5:**
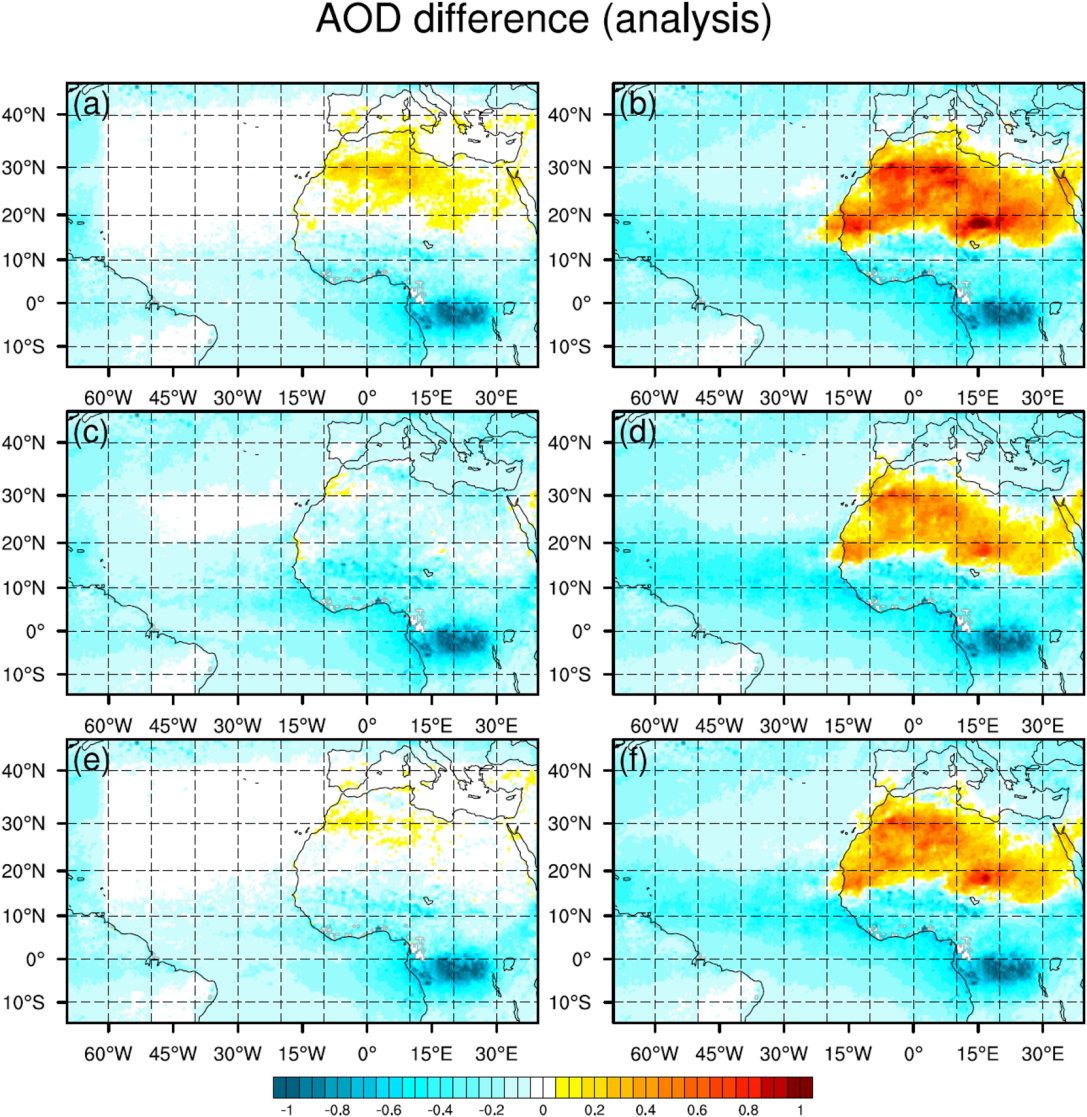
Mean differences of AOD analyses of (a) 3DVAR_ALL, (b) 3DVAR_NoAOD, (c) EnKF_ALL, (d) EnKF_NoAOD, (e) Hybrid_ALL, and (f) Hybrid_NoAOD from MODIS AOD observations.

**Figure 6:**
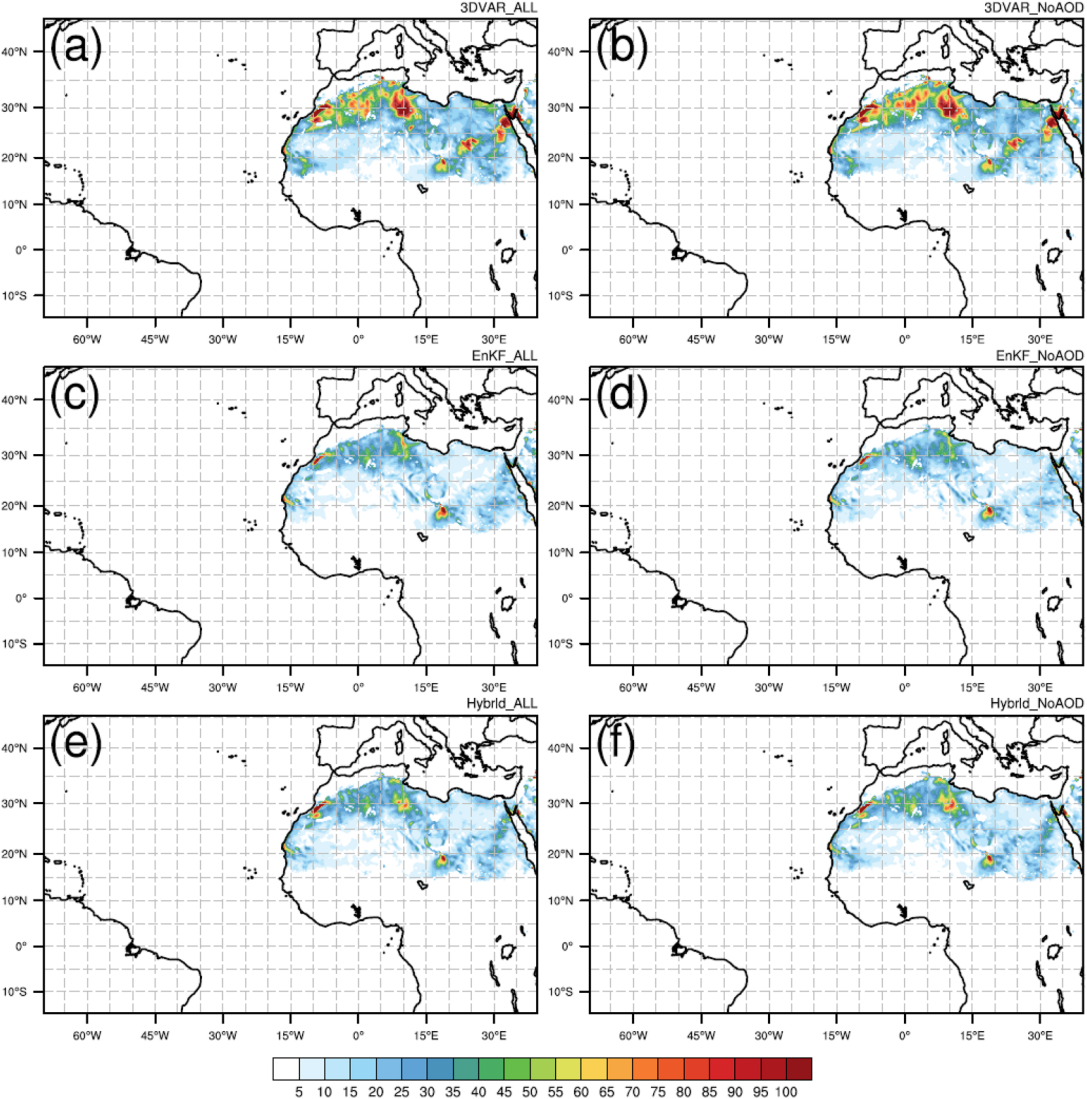
Monthly‐average total dust emission fluxes (i.e., sum over all five size bins; μg m^−2^ s^−1^) for (a) 3DVAR_ALL, (b) 3DVAR_NoAOD, (c) EnKF_ALL, (d) EnKF_NoAOD, (e) Hybrid_ALL, and (f) Hybrid_NoAOD.

Figures 4a–4f show the average of all AOD analyses for each experiment. The overall spatial pattern of the observed AOD distribution (Figure 4h) is well captured by all DA experiments. However, in the three “NoAOD” experiments, AOD values are overestimated (underestimated) over North Africa (Atlantic Ocean) compared to the observations (see Figures 5b, 5d, and 5f). Through AOD assimilation, AOD analysis values over North Africa are reduced, while those over the Atlantic Ocean are increased, as demonstrated by the results of the three “ALL” experiments (also see Figures 5a, 5c, and 5e). To identify whether the improved AOD analysis over North Africa in “ALL” experiments is due to the assimilation of AOD or meteorology data, monthly averaged total (i.e., sum of five size bins) dust emission fluxes from six DA experiments are plotted (Figure 6). Dust emission flux of the “ALL” experiment is similar to that of the corresponding “NoAOD” experiment for each DA method. This implies that the AOD assimilation mainly contributes to the improved AOD analysis in the “ALL” experiments compared to the “NoAOD” experiments. Nevertheless, the AOD analyses from the “ALL” experiments still differ significantly from observations in several areas. For example, analysis AOD values exceed observations over Algeria and are smaller than the observed values over central Africa (between 0°N and 15°N) in 3DVAR_ALL. In the EnKF_ALL experiment, analysis and MODIS AOD values agree quite well over Algeria, but analysis AOD values are generally smaller than observed over the Atlantic Ocean. Both 3DVAR_ALL's larger‐than‐observed AOD values over Algeria and EnKF_ALL's smaller‐than‐observed AOD values over the Atlantic disappear in analyses of Hybrid_ALL, which gives the best result (Figure 5). Domain‐averaged monthly‐mean differences and RMSDs between the AOD analyses from all six experiments and MODIS AOD observations are calculated to facilitate a quantitative comparison. The differences (RMSDs) for 3DVAR_ALL, 3DVAR_NoAOD, EnKF_ALL, EnKF_NoAOD, Hybrid_ALL, and Hybrid_NoAOD are −0.019 (0.131), −0.022 (0.401), −0.132 (0.192), −0.107 (0.349), −0.044 (0.109), and −0.079 (0.360), respectively. The AOD analyses of Hybrid_ALL best agree with MODIS AOD observations in terms of the RMSD, while the analyses of 3DVAR_ALL are most similar to MODIS observations in terms of simple difference. Note that the latter result is due to the presence of large areas of positive and negative differences of similar magnitude, rather than high agreement between 3DVAR analyses and MODIS observations. The AOD distributions of the three “NoAOD” experiments differ substantially from each other due to differences in the analyzed wind fields for each experiment. These wind differences produce differences in dust emission (Figures 6b, 6d, and 6f), which is a function of the 10‐m wind speed, ultimately leading to differences in the simulated amount and distribution of dust aerosol. Consistent features with mean AOD discrepancy distributions can be found in AOD RMS difference distributions (figure not shown).

**Figure 7:**
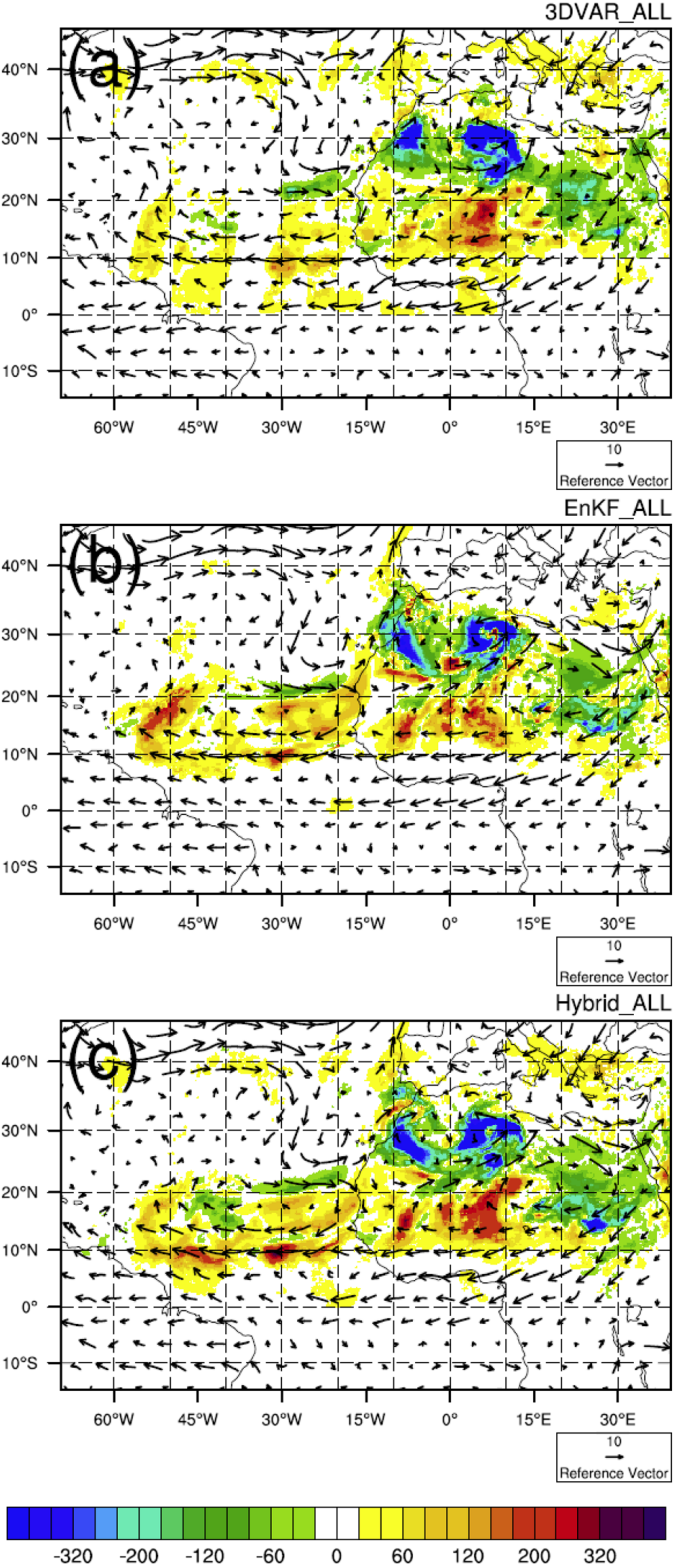
Analysis increments of 700‐hPa total dust mixing ratio (μg kg^−1^) at 1200 UTC 4 June 2015 for (a) 3DVAR_ALL, (b) EnKF_ALL, and (c) Hybrid_ALL experiments.

Analysis increments of 700‐hPa total dust mixing ratio at 12 UTC 4 June 2015 from the three “ALL” experiments are shown in Figure 7. In the summer, the midlevel (~700‐hPa) flow over North Africa and the East Atlantic is often dominated by the African easterly jet and associated waves. Overall, analysis increments of the EnKF_ALL and Hybrid_ALL experiments better reflect the background flow than those of the 3DVAR_ALL experiment. For example, the positive dust increments over the Atlantic between 10°N and 20°N are more longitudinally continuous in EnKF_ALL and Hybrid_ALL—coinciding with the African easterly jet originating over the Sahara Desert—than in 3DVAR_ALL. In short, flow‐dependent BECs are more efficient in extracting information from the observations than static BECs, which is the primary reason that Hybrid_ALL produces AOD analyses that best match the observations.

In summary, comparing the monthly‐mean AOD analyses for the “ALL” and “NoAOD” experiments to observations shows better agreement between the “ALL” monthly‐mean AOD analyses and observations. Of the three “ALL” experiments, Hybrid_ALL produces the monthly‐mean AOD analysis that best matches the observations, while the AOD analysis from EnKF_ALL performs better than that from 3DVAR_ALL. These results demonstrate that while using flow‐dependent BECs alone can be effective for improving AOD assimilation, additional gains can be attained using static and flow‐dependent BECs simultaneously.

#### 4.1.2 Comparison With AERONET AOD Observations

**Figure 8:**
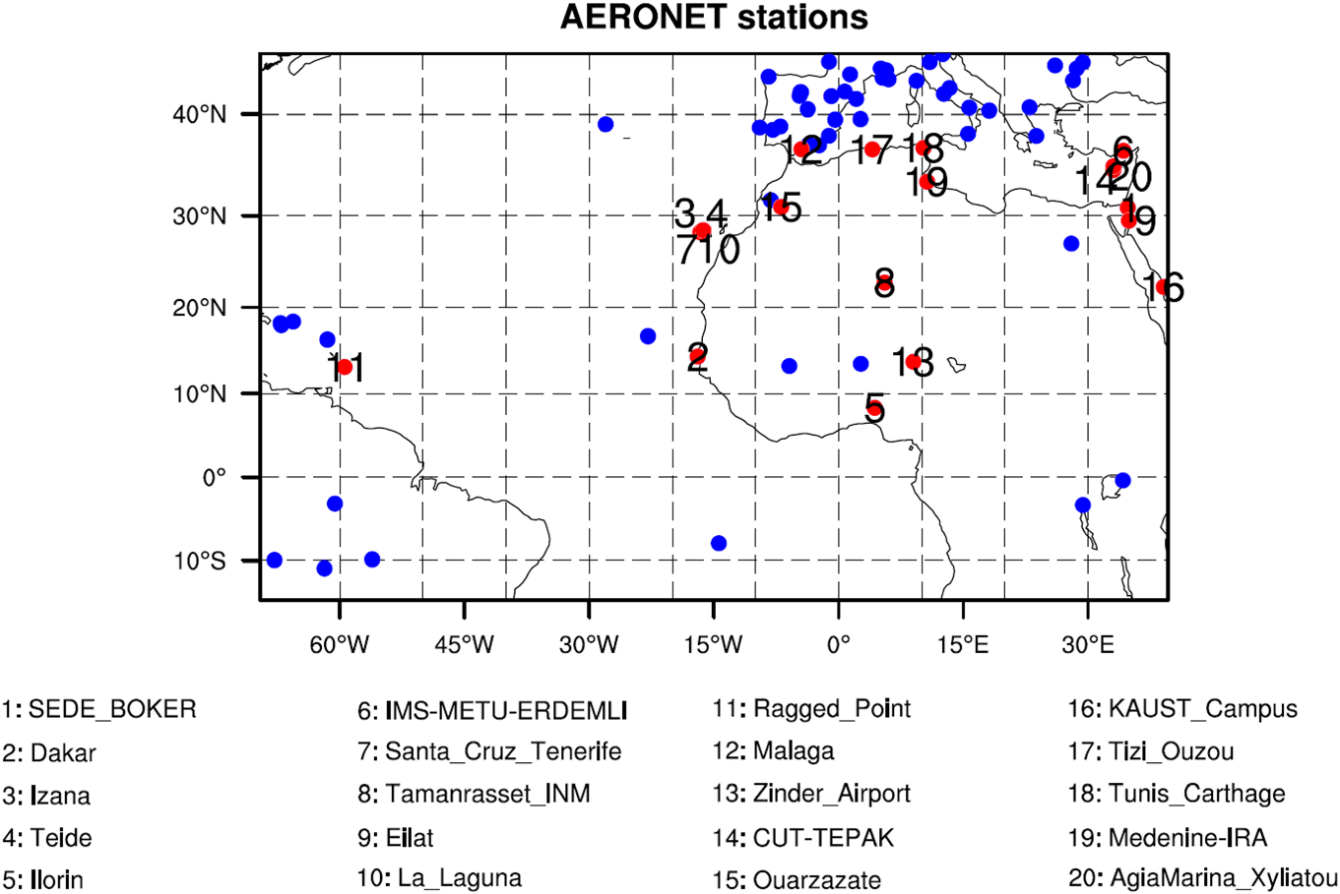
Geographical locations of AERONET stations over Domain 1. AERONET stations used for verification are denoted by red dots, and their station names are given.

AERONET AOD observations (Cuevas et al., 2019; Giles et al., 2019) represent an independent data set that can be used to assess our AOD analyses and forecasts. The AERONET is a ground‐based aerosol observation network established by NASA and PHOTONS. AERONET observations provide globally distributed, high‐temporal frequency observations of AOD at several wavelengths (Jiang et al., 2019). Version 3 AERONET data have three data quality levels: Level 1.0 (unscreened), Level 1.5 (cloud screened and quality controlled), and Level 2.0 (quality assured). In this study, only Level 2.0 AERONET AOD data at 500 nm are used for AOD verification. Note that AOD values at 550 nm can be transformed to 500 nm using the AE (Eck et al., 1999) but the AOD difference between the two wavelengths is not large for a range of AE values. We selected 20 of the 74 AERONET stations found within Domain 1, located in North Africa, the Atlantic Ocean, and the Arabian Peninsula, for AOD verification (Figure 8). Since AERONET AOD data, when available, are reported far more frequently than the 6‐hr analysis interval but not necessarily exactly at each analysis time, the AERONET AOD values used to evaluate our AOD analyses are computed by averaging all valid data within a 2‐hr window centered at each analysis time.

**Figure 9:**
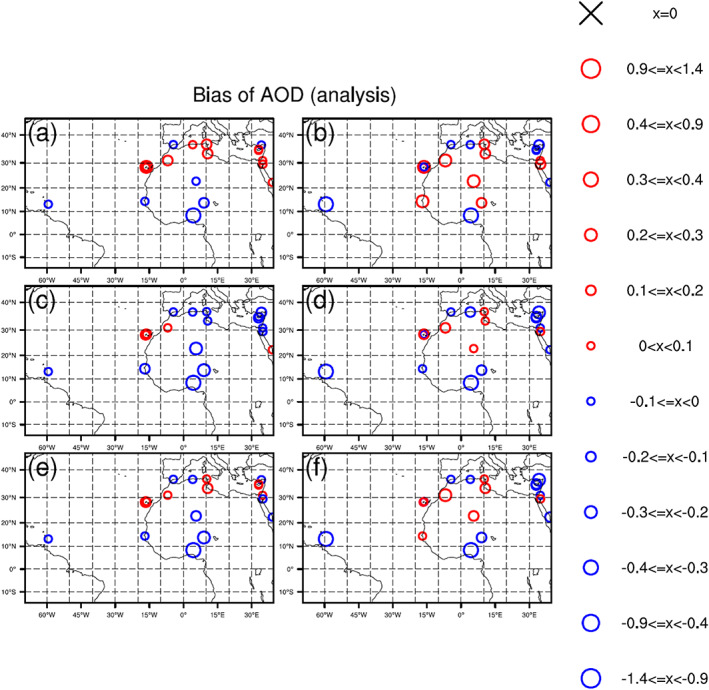
Biases of AOD for analyses of (a) 3DVAR_ALL, (b) 3DVAR_NoAOD, (c) EnKF_ALL, (d) EnKF_NoAOD, (e) Hybrid_ALL, and (f) Hybrid_NoAOD experiments. Biases are calculated against AERONET AOD observations.

Figure 9 shows the AOD biases for all six DA experiments relative to AERONET observations. Biases at each AERONET station and for each experiment are computed by averaging differences between AERONET and analysis AOD values over 120 DA cycles. For the three “NoAOD” experiments (Figures 9b, 9d, and 9f), AOD analyses have positive biases at almost all AERONET stations over North Africa and negative biases at stations in Spain, Algeria, Nigeria, Cyprus, and the West Atlantic. Positive biases are largest for the 3DVAR_NoAOD experiment.

Compared to the AOD analyses for the “NoAOD” experiments, those for the corresponding “ALL” experiments have smaller AOD bias magnitudes at most AERONET sites. This includes bias magnitude reductions at stations outside of Nigeria where “NoAOD” analyses are negatively biased and at all stations where “NoAOD” analyses are positively biased (Figures 9a, 9c, and 9e). Sometimes the magnitudes of “ALL” and “NoAOD” are the same, and some positively biased locations in the “NoAOD” analyses show negative biases in the “ALL” analyses. For the 3DVAR_ALL experiment, AOD analyses at stations between 25°N and 35°N are positively biased, while AOD analyses from the EnKF_ALL experiment are negatively biased at most stations. Biases in the Hybrid_ALL AOD analyses lie between those from the 3DVAR_ALL and EnKF_ALL experiments. The pervasiveness of negative biases over broad regions in the EnKF_ALL analyses is partly attributed to the use of the ensemble mean—which tends to reduce variations present in individual members—for bias calculation.

The AERONET‐based AOD comparison described in this section is consistent with the MODIS‐based AOD comparison in section 4.1.1.

#### 4.1.3 Comparison With CALIOP Backscatter Observations

The vertical distribution of dust aerosol is important because the effects of dust particles on radiation and clouds can vary depending on the altitude of a dust layer relative to clouds in the same atmospheric column. The CALIOP instrument aboard the CALIPSO satellite provides invaluable, detailed vertical profiles of clouds and aerosols along its track (Young et al., 2018). Total (Level 2 [L2]) aerosol backscatter information from CALIOP is used to evaluate the vertical distribution of dust in the analyses and forecasts from all experiments. This data set, which represents the retrieved backscatter due solely to aerosols, is chosen for this purpose since it allows an evaluation of modeled dust data without requiring a simultaneous assessment of other nonaerosol model variables (i.e., for clouds, absorbing gases, and others), as would be required if the Level 1 attenuated backscatter data set were chosen instead. Additionally, CALIOP aerosol backscatter observations are suitable for model validation since, as with AERONET data, they represent an independent source of aerosol information.

To validate model analyses against CALIOP total aerosol backscatter data, the modeled dust distribution is first transformed into CALIOP‐equivalent backscatter values along CALIPSO tracks using the Goddard Satellite Data Simulator Unit (G‐SDSU; Matsui et al., 2014) for each DA experiment. Both CALIOP and the model‐simulated total backscatter data are then processed and interpolated to the same resolution (from finer to coarser) according to the procedure outlined below:

1 Flag cloud‐contaminated and unreliable CALIOP L2 total backscatter data (CAL_b) by

- selecting data points that are classified as “aerosol” or “clear air” by the CALIOP L2 vertical feature mask (VFM) product
- using only data points with extinction QC flag values of 0, 1, 2, 16, 18, or 32,768 (Toth et al., 2018).

Note that the vertical resolution of both CALIOP L2 VFM and extinction QC data is finer (30 m) than that of CALIOP L2 total backscatter data (60 m), meaning that two VFM and extinction QC values exist for each backscatter value. Thus, a backscatter value is considered acceptable if both of its corresponding VFM values indicate “aerosol” or “clear air” and both of its associated extinction QC flag values are one or more of those listed in (b) above.

2 Assign a small backscatter value (10^−4^ km^−1^ sr^−1^) to all “clear‐air” data points in CAL_b, to represent the backscatter from “background” aerosols that are present even when CALIOP fails to detect a significant aerosol feature.
2. Interpolate G‐SDSU‐simulated backscatter (GSDSU_b) data horizontally and vertically to the grid used by CAL_b.
4 Exclude corresponding data points in both CAL_b and GSDSU_b where

- CAL_b values are equal to the background value (CAL_b = 10^−4^ km^−1^ sr^−1^) and total modeled dust mixing ratios at the same points are <1 μg m^−3^. This prevents data points with small background aerosol amounts from causing differences between CALIOP and model backscatter (i.e., bias), as well as backscatter differences between experiments, to appear misleadingly small.
- CAL_b data are flagged as cloud contaminated or unreliable.
- Interpolated GSDSU_b data are invalid.

5 Compute CAL_b and GSDSU_b values on model vertical levels by averaging valid data.

**Figure 10:**
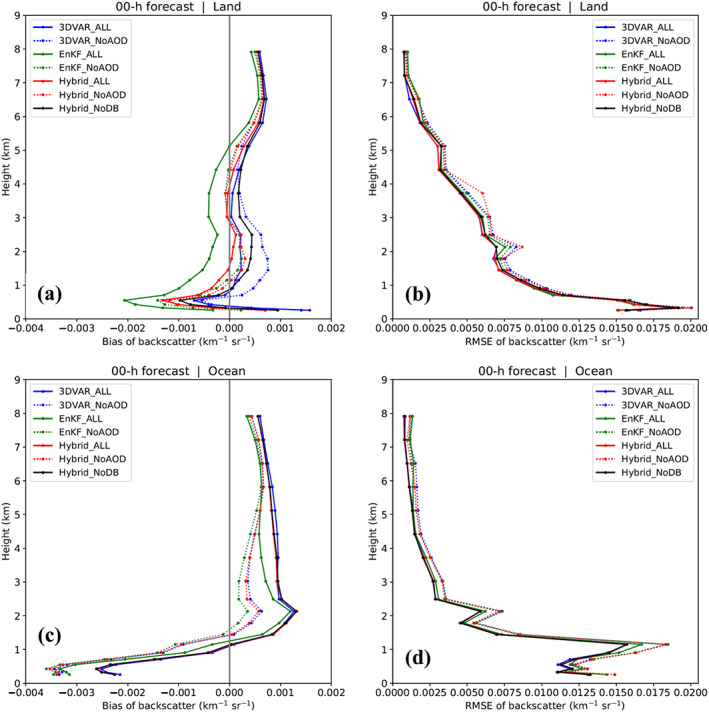
Vertical distributions of (a and c) bias and (b and d) RMSE of backscatter (km^−1^ sr^−1^) for analyses. CALIOP backscatter observations whose paths cross over (a and b) land and (c and d) ocean are used for bias and RMSE calculation.

Two average vertical profiles of total backscatter biases are computed for each experiment: one for data points over North Africa (i.e., land; Figure 10a) and the other for data points over the Atlantic (i.e., ocean; Figure 10c). While magnitudes differ between experiments, all bias profiles for the same surface type (land or ocean) share the same general shape. However, bias profiles for land and ocean are noticeably different in both magnitude and shape. Comparing the backscatter analyses of the “ALL” experiments to those of the corresponding “NoAOD” experiments over land shows that AOD assimilation in (1) 3DVAR_ALL improves and degrades the analysis backscatter profile between 0.5 and 4.5 km and above 4.5 km, respectively; (2) EnKF_ALL improves and degrades the analysis backscatter profile above and below 5 km, respectively; and (3) Hybrid_ALL improves the analysis backscatter profile between 1.5 and 3.5 km but degrades it elsewhere. In short, AOD assimilation appears to reduce backscatter analysis biases at most levels but increases them at others. This is due to the general reduction in column AOD values caused by AOD assimilation, consistent with the results of sections 4.1.1 and 4.1.2. Since MODIS AOD values reflect the extinction of visible light due to all aerosols in an atmospheric column, an assimilation‐driven reduction in column‐total AOD at a horizontal model grid point is the result of reduced modeled dust concentrations throughout the column where such concentrations are initially non‐zero. This produces smaller total backscatter values and the general leftward shift seen for the “ALL” land‐based average analysis backscatter bias profiles relative to those of the “NoAOD” experiments. In general, the land‐based backscatter bias profiles for the “ALL” experiments show that Hyrbid_ALL produces better backscatter analyses than 3DVAR_ALL below 0.25 km and above 1.5 km but not in between, while EnKF_ALL produces worse backscatter analyses than both Hybrid_ALL and 3DVAR_ALL at all levels below 5 km.

RMSEs of the “ALL” analyses over land are slightly smaller than those of their “NoAOD” counterparts at nearly all levels (Figure 10b), in contrast with the biases for the two sets of experiments. The differences in RMSE and bias values between the “ALL” and “NoAOD” experiments, together with the fact that RMSE magnitudes are one order larger than corresponding bias magnitudes, imply that modeled backscatter values are not consistently greater or less than observed values at any given level. As a result, backscatter biases are small and do not adequately reflect the small positive effect that AOD assimilation has on the vertical dust distribution, which is illustrated through RMSEs. Of the three “ALL” experiments, 3DVAR_ALL and Hybrid_ALL have slightly smaller RMSEs than does EnKF_ALL, especially at middle to upper levels.

Although AOD values are generally smaller over the Atlantic than over North Africa, backscatter biases and RMSEs are not (i.e., larger percentage biases). For instance, a negative bias peak appears near the surface over both land and ocean, but the magnitude of the peak over ocean is almost twice larger than that over land (Figure 10c). One reason that the low‐level negative backscatter bias peak is particularly large over the ocean is the lack of sea salt aerosols in our model, which can nevertheless contribute significantly to observed backscatter values. Since AOD values are generally underestimated over the ocean without AOD assimilation (Figure 5), AOD DA has the effect of shifting backscatter bias profiles to the right, which reduces the negative biases below 1.5 km but increases positive biases above. Ultimately, this is due to the fact that AOD observations do not provide any information regarding the vertical distribution of aerosols. To improve modeled dust (and thus backscatter) profiles over the ocean, either that the model background data provided as input to GSI must include marine aerosols, or that aerosol observations with vertical information, such as CALIOP backscatter, must be assimilated. Exploring these approaches is beyond the scope of this work and is left for future investigation.

As with RMSEs over land, RMSEs over ocean are reduced slightly at nearly all levels after AOD assimilations (Figure 10d). Near their ~1‐km peak, RMSEs are greater for EnKF_ALL than for 3DVAR_ALL and Hybrid_ALL.

In conclusion, AOD assimilation reduces aerosol backscatter RMSEs slightly at nearly all levels, with better performance from the variational and hybrid methods than the EnSRF method. The slightly worse performance for the EnSRF method is due in part to (1) the use of ensemble mean (smoothness) in evaluating the EnKF analyses and (2) the use of the adjoint operator in both the 3D‐Var and hybrid methods. While AOD assimilation can reduce average AOD analysis biases with respect to both MODIS and independent, nonassimilated AERONET data, its impact on backscatter bias profiles is less clear and depends on the DA method used and profile height. This is due to the fact that AOD data do not describe the vertical distribution of aerosols. Improving backscatter bias and RMSE profiles thus requires the assimilation of observations that can describe the vertical distribution of aerosols, such as CALIOP backscatter, which is expected to have a positive impact on AOD RMSEs as well.

### 4.2 Forecast Results

Similar to the analysis evaluation procedure above, dust forecasts are verified by comparing model results against MODIS AOD, AERONET AOD, and CALIOP backscatter observations. Taylor diagrams, which can provide concise statistical summaries of how well two patterns (i.e., model forecasts and observations) match each other in terms of their correlation, their RMS difference, and the ratio of their variances (Taylor, 2001), are introduced in this section to present and assess forecast results. Since two‐way interaction is used during the forecasts, the results from a high‐resolution child domain overwrite the results for the same area in its mother domain. Thus, in this section, results from Domain 1 are used for verification in order to cover a larger area.

#### 4.2.1 AOD Forecasts

##### 4.2.1.1 Comparison With MODIS AOD Observations

**Figure 11:**
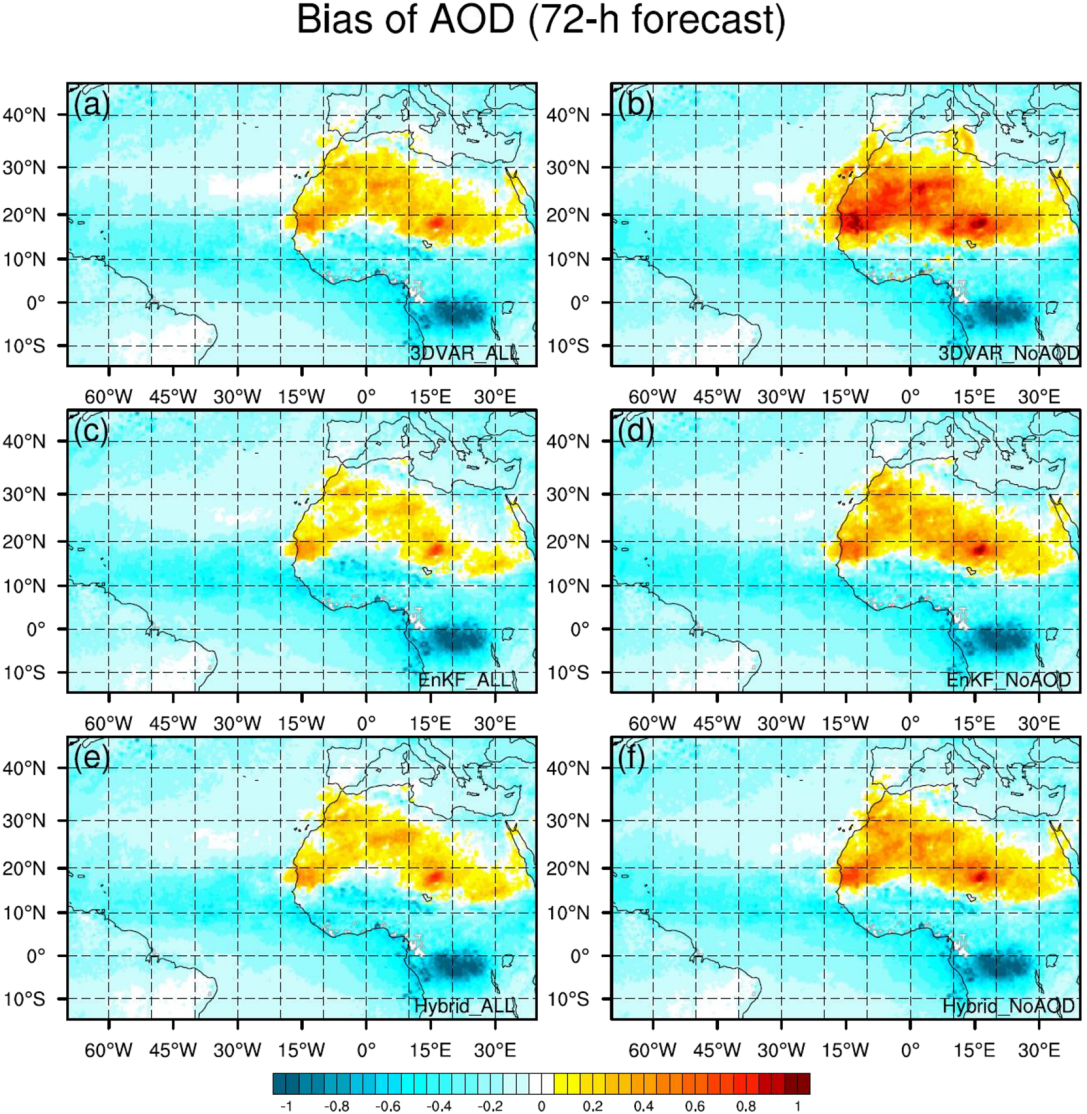
The same as Figure 5 but for 72‐hr forecasts.

The spatial pattern of AOD forecast biases with respect to MODIS AOD is similar for all six experiments at 24, 48, and 72 hr, as shown in Figure 11 for the 72‐hr forecast, with positive biases over the deserts of North Africa and negative biases elsewhere. The patterns/magnitudes of the “NoAOD” 72‐hr AOD forecast biases are very similar to those of the corresponding analysis biases (Figure 5). For the “ALL” experiments, AOD bias magnitudes grow with increasing forecast length while differences between the three experiments shrink. This behavior is caused by the natural growth of systematic errors during the free forecast following the abrupt change in the AOD distribution due to AOD DA.

**Figure 12:**
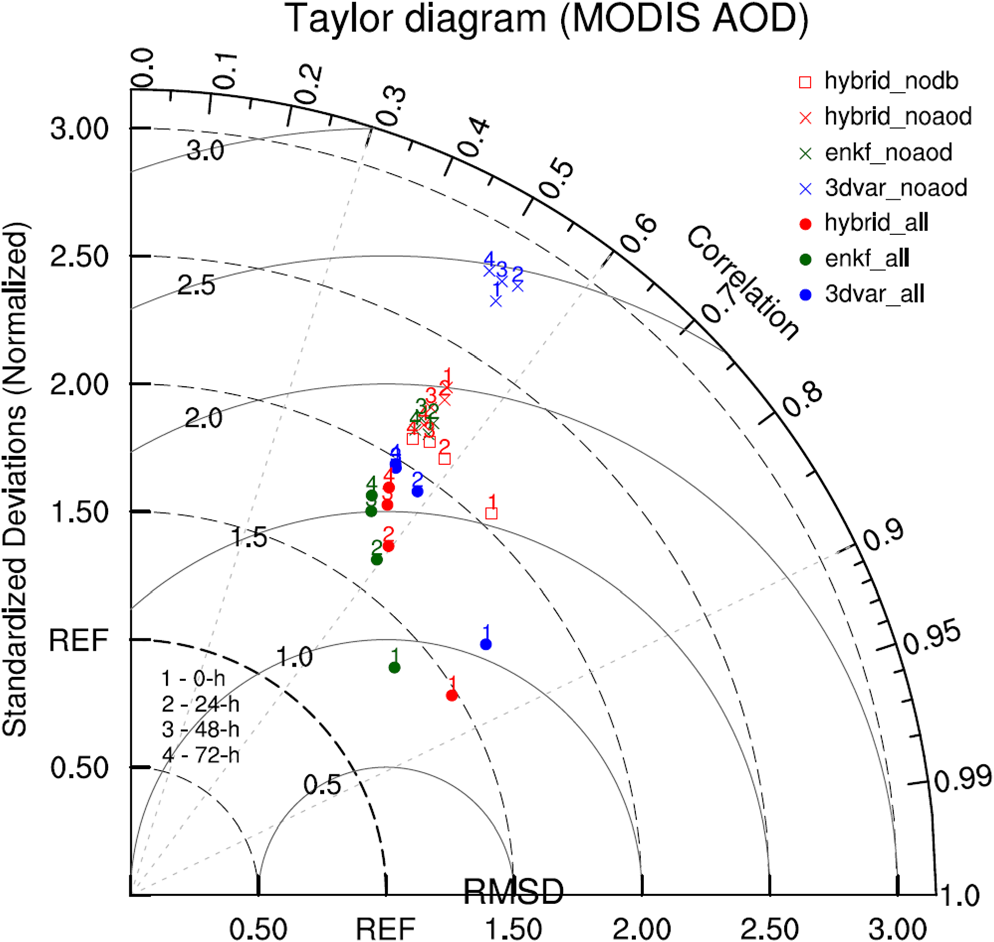
Taylor diagram for 0‐, 24‐, 48‐, and 72‐hr AOD forecasts of seven DA experiments. Blue, green, and red dots denote 3DVAR_ALL, EnKF_ALL, and Hybrid_ALL experiments, respectively. Blue, green, and red crosses indicate 3DVAR_NoAOD, EnKF_NoAOD, and Hybrid_NoAOD experiments, respectively. Red square denotes Hybrid_NoDB experiment. The numbers 1, 2, 3, and 4 correspond to 0‐, 24‐, 48‐, and 72‐hr forecasts, respectively.

Figure 12 shows a Taylor diagram for the 0‐, 24‐, 48‐, and 72‐hr AOD forecasts from all six DA experiments. Overall, the “ALL” AOD forecasts are in better agreement with MODIS observations than the “NoAOD” AOD forecasts in all aspects (i.e., “ALL” forecasts show higher correlations, similar spatial variability to the observation, and smaller RMS differences), with the exception of the 72‐hr forecasts when all experiments have a similar correlation. AOD forecasts from the “ALL” experiments worsen with increasing forecast length in terms of AOD RMS difference, spatial correlation, and normalized standard deviation. Taylor statistics for the “NoAOD” forecasts, on the other hand, change only slightly, causing the Taylor statistics for the “ALL” and “NoAOD” experiments (other than 3DVAR_NoAOD) to converge with increasing forecast length. Of the “ALL” experiments, Hybrid_ALL and EnKF_ALL produce the best AOD analysis and AOD forecasts (at 24, 48, and 72‐hr), respectively, the latter of which are slightly better than the corresponding forecasts of Hybrid_ALL.

In summary, due to AOD assimilation, the AOD forecasts of the “ALL” experiments outperform those of the “NoAOD” experiments compared to observations. However, differences in forecast quality between the “ALL” and “NoAOD” experiments, as well as among the three “ALL” experiments, shrink as forecast length increases. At the initial (analysis) time, modeled AOD values from Hybrid_ALL best match the observations, while the best AOD forecasts are produced by EnKF_ALL, which hold a slight advantage over their Hybrid_ALL counterparts from a Taylor statistics perspective.

##### 4.2.1.2 Comparison With AERONET AOD Observations

**Figure 13:**
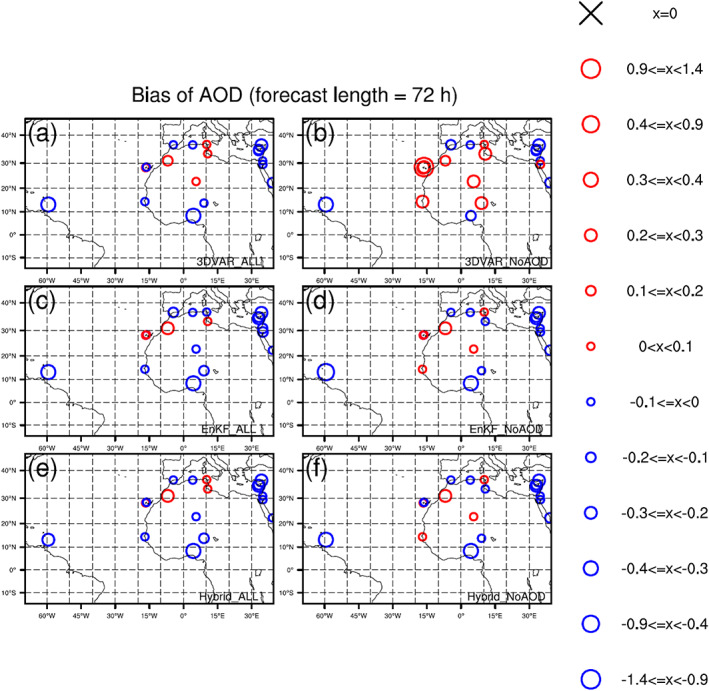
The same as Figure 9 but for 72‐hr forecasts.

The 72‐hr AOD forecast biases at each AERONET station from all six DA experiments are shown in Figure 13. Forecast biases for the three “NoAOD” experiments are larger than those of the corresponding “ALL” experiments. Differences among the “ALL” experiments become smaller with forecast length (results for 24‐ and 48‐hr forecasts are not shown). The 72‐hr AOD forecast biases with respect to AERONET observations are consistent with those computed with respect to MODIS observations.

**Figure 14:**
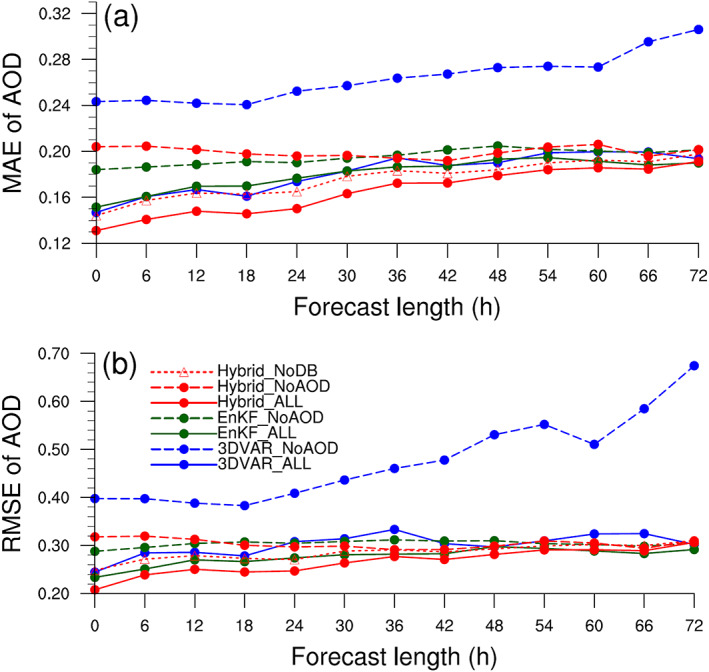
Domain‐averaged (a) MAE and (b) RMS error (RMSE) of AOD as a function of forecast length. MAE and RMSE are calculated using AOD values from selected 20 AERONET stations.

Figure 14 shows AOD forecast mean absolute errors (MAEs) and RMSEs as a function of forecast length averaged over 20 AERONET stations. AOD MAEs of the “ALL” experiments are smaller than those of the corresponding “NoAOD” experiments regardless of DA methods. AOD forecast improvement through the AOD assimilation is more noticeable when the 3D‐Var method is used (~35% to 60%). Of the “ALL” experiments, forecasts of the Hybrid_ALL are better than those of the 3DVAR_ALL and EnKF_ALL regardless of forecast lengths (Figure 14a).

AOD RMSE values increase in all experiments other than EnKF_NoAOD and Hybrid_NoAOD, and, with the exception of 3DVAR_NoAOD, tend to converge with increasing forecast length (Figure 14b). Using the 3D‐Var method, AOD assimilation reduces AOD RMSEs of subsequent forecasts at all times, with RMSE reductions becoming more substantial with increasing forecast length. For the EnSRF and hybrid methods, AOD assimilation improves forecast AOD RMSEs for the first 48 hr, after which time RMSE reductions become less significant. These results clearly show that AOD assimilation positively impacts AOD analyses and forecasts.

Regardless of whether or not AOD data are assimilated, the EnSRF and hybrid methods produce better dust forecasts than the 3DVAR method. When AOD data are assimilated, RMSEs indicate that the hybrid method produces the best AOD forecasts, while the EnSRF and 3D‐Var methods produce the second best and worst AOD forecasts, respectively.

**Figure 15:**
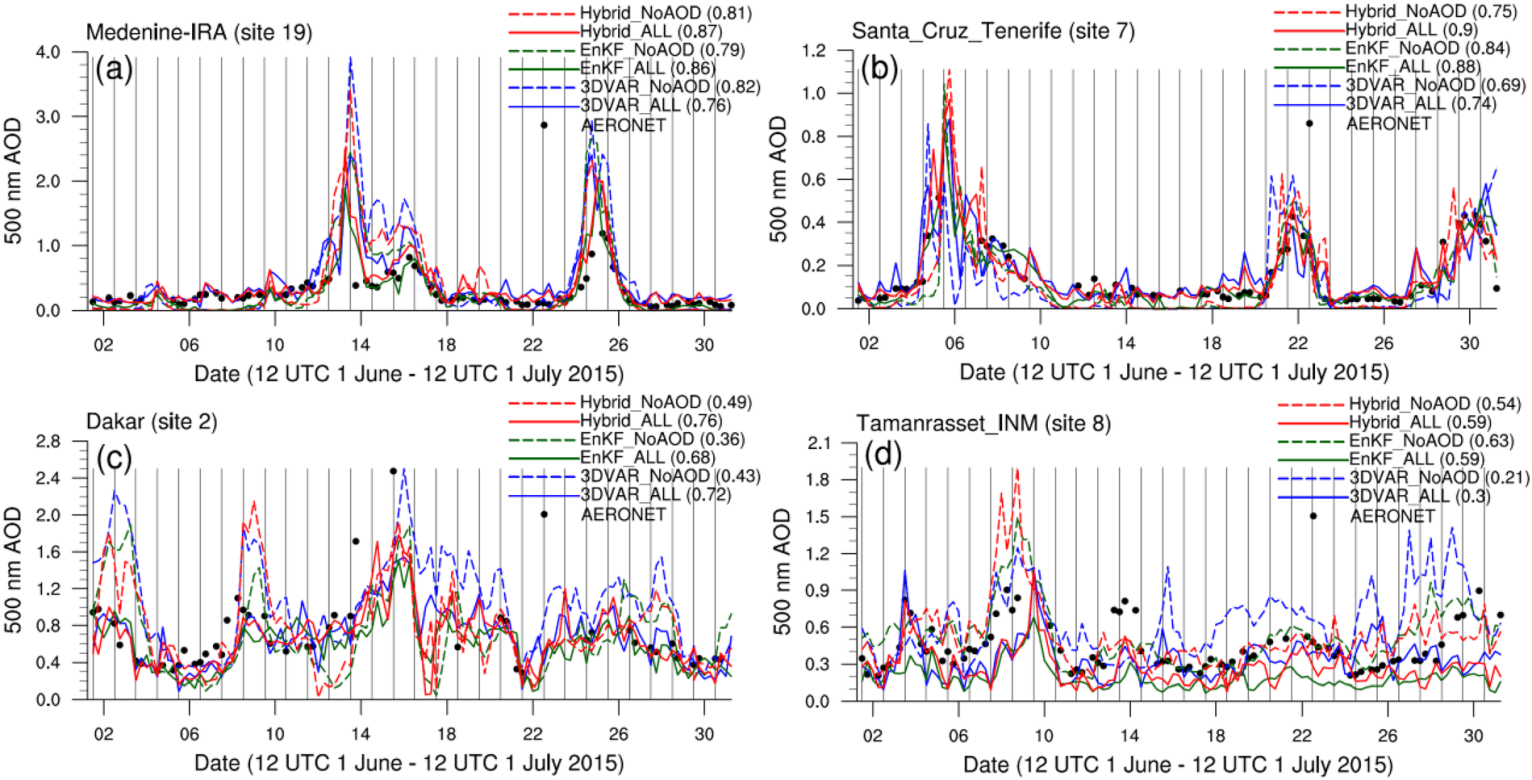
Six‐hourly time series of AOD from 1200 UTC 1 June 2015 to 1200 UTC 1 July 2015 at (a) Medenine‐IRA, (b) Santa Cruz, Tenerife, (c) Dakar, and (d) Tamanrasset_INM AERONET stations. Correlation value between time series of AERONET observation and each DA experiment is shown next to the name of each experiment.

While our AOD forecast assessments have thus far focused on month‐long averages, it is also important to determine how well each experiment captures short‐term, day‐to‐day fluctuations in AOD. To do this, 0‐, 6‐, 12‐, and 18‐hr AOD forecasts from each experiment starting from each day's 1200 UTC DA cycle, when most MODIS AOD observations are assimilated in the “ALL” experiments (Figure 1), are stitched together and interpolated to AERONET stations to form a month‐long AOD time series for each station that can be compared to the station's own AOD time series. Of the 20 selected stations within Domain 1, four stations in North Africa (Medenine‐IRA, Santa Cruz, Dakar, and Tamanrasset_INM; see Figure 8 for their locations) are selected for comparison since they are located near or within the main region of interest and have fewer missing values than others in the same area. At Medenine‐IRA, which is located in Tunisia, all AOD time series, both simulated and observed, show two peaks near 1200 UTC on 17 and 25 June. Although forecasted AOD values exceed observations near these peaks, observed and forecasted AODs over the entire 1‐month period are well correlated for all experiments, with correlation coefficients between the two exceeding 0.75 for all experiments. Of the three “ALL” experiments, Hybrid_ALL has the highest correlation coefficient (0.87), followed by that of EnKF_ALL, and finally 3DVAR_ALL (Figure 15a). At Santa Cruz on the island of Tenerife, AERONET‐observed high‐AOD events on 6, 22, and 30 June are well captured in all experiments. High correlation between observed and forecasted AOD is again found at this station for all experiments (correlation coefficients above 0.7), with higher correlation for the “ALL” experiments than the corresponding “NoAOD” experiments. As with the Medenine‐IRA station data, Hybrid_ALL produces the best forecast‐observation correlation at this station (Figure 15b). At the Dakar station in Senegal, large observed temporal fluctuations in AOD are accurately captured in all experiments but are represented particularly well by the “ALL” experiments, whose correlation coefficients are greater than those of the corresponding “NoAOD” experiments. Hybrid_ALL once again has the highest correlation, followed by 3DVAR_ALL and EnKF_ALL (Figure 15c). Finally, at the Tamanrasset_INM station in Algeria, some observed peaks in AOD near 14 and 30 June are not found in the forecasts, resulting in lower correlation coefficients (below 0.65). The “EnKF” and “Hybrid” experiments have higher correlation coefficients than the “3DVAR” experiments do (Figure 15d).

In short, the validation of AOD forecasts against AERONET observations reinforces the results of similar validation efforts against MODIS observations: AOD assimilation clearly improves AOD analyses and subsequent forecasts, particularly for the 3D‐Var method. Comparisons with AERONET data show that the benefits of AOD assimilation persist for about 48 hr for the EnSRF and hybrid methods. The forecasts of Hybrid_ALL perform the best in terms of monthly averaged bias and correlation with observed day‐to‐day AOD fluctuations, followed by the forecasts of EnKF_ALL, and finally 3DVAR_ALL. Hybrid_ALL's superiority with respect to AERONET data is attributable to the use of the hybrid DA method, which enjoys the benefits of flow‐dependent BECs while minimizing potential issues associated with insufficient ensemble spread by using static BECs as well. Note, however, that it is EnKF_ALL, not Hybrid_ALL, that performs the best with respect to MODIS AOD data. This could be due in part to large differences in spatial coverage and temporal resolution (2‐hr average for observations vs. snapshot data for model) between AERONET and MODIS AOD observations.

#### 4.2.2 Total Aerosol Backscatter Profiles

**Figure 16:**
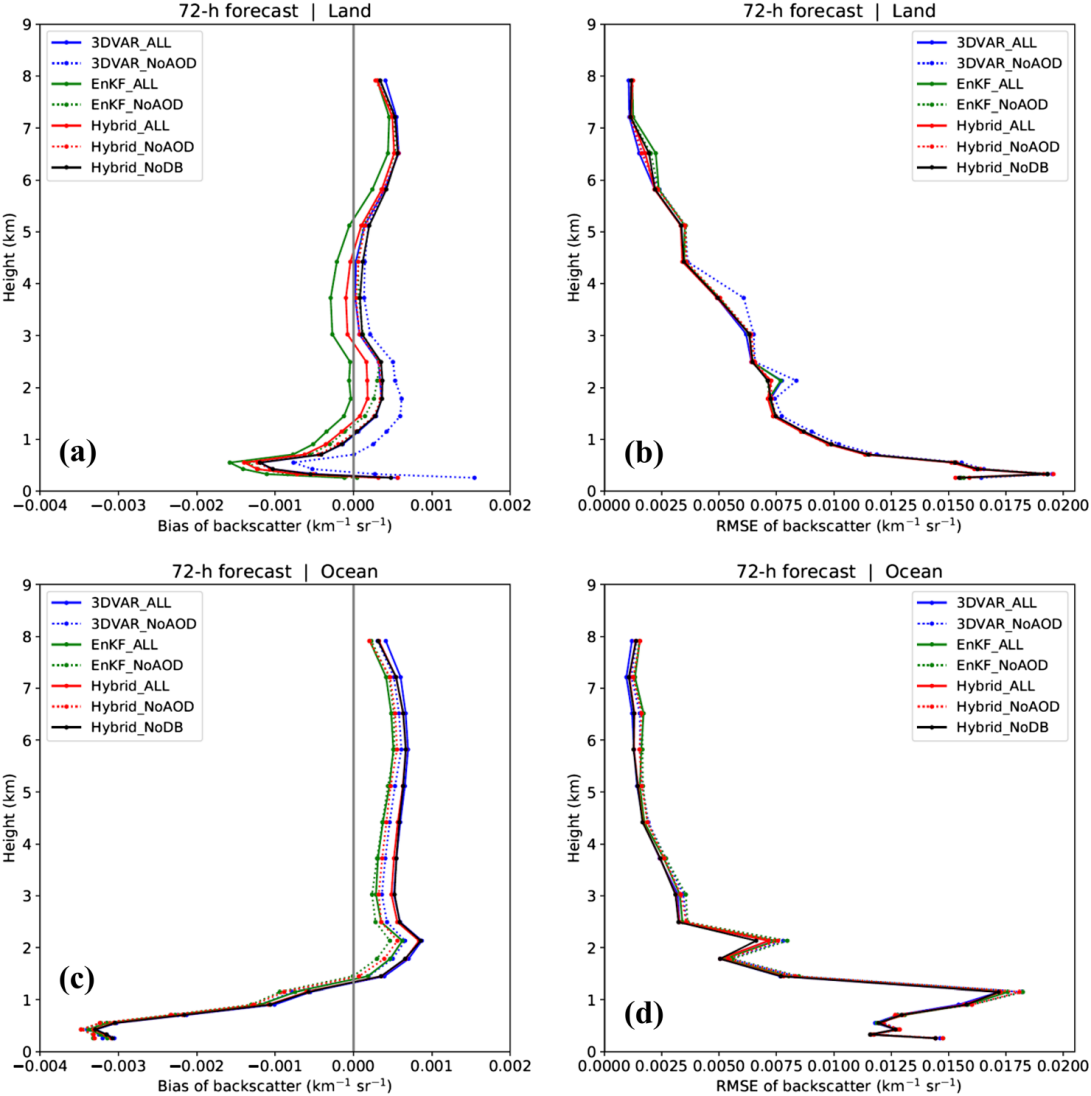
The same as Figure 10 but for 72‐hr forecasts.

Forecasted total backscatter profiles are compared with those from CALIOP L2 observations. Mean 72‐hr forecast biases and RMSEs with respect to CALIOP data are presented in Figure 16. Over land, the shape of each experiment's 72‐hr forecasted backscatter bias profile (Figure 16a) is generally similar to that of its analysis profile (Figure 10a). However, there are fewer differences among the bias profiles of all experiments at the 72‐hr forecast time. The effect of AOD assimilation on 72‐hr forecast bias profiles depends significantly on the DA method. For 3D‐Var, AOD assimilation reduces bias magnitudes at all levels other than between 0.25 and 1 km and above 7 km, compared to 3DVAR_NoAOD. For the hybrid method, forecasted backscatter bias magnitudes are reduced by AOD assimilation below 0.25 km and above 1.25 km and degraded elsewhere, compared to Hybrid_NoAOD. Finally, for EnKF, AOD assimilation reduces forecast bias magnitudes between 1.5 and 3.0 km and above 5.0 km, compared to EnKF_NoAOD.

Over the ocean, differences among the backscatter bias profiles from all experiments (Figure 16c) are also smaller at the 72‐hr forecast time than the analysis time (Figure 10c). The forecast bias profiles share the same basic features as their analysis bias counterparts, with large negative biases below 1 km and positive biases above. Additionally, as with the analysis bias profiles, AOD assimilation causes the 72‐hr forecast bias profiles to shift rightward toward larger values (smaller negative or larger positive biases), but the shift is small relative to that seen in the analyses. Backscatter RMSE profiles for the 72‐hr forecasts and their analyses are also similar over both land and ocean for all experiments, although RMSE differences among experiments are smaller for the forecasts (Figures 16b and 16d). We again emphasize that bias magnitudes are about one order smaller than RMSE values for both the analyses and forecasts due to the cancelation of positive and negative biases that occurs when averaging individual bias profiles together.

**Figure 17:**
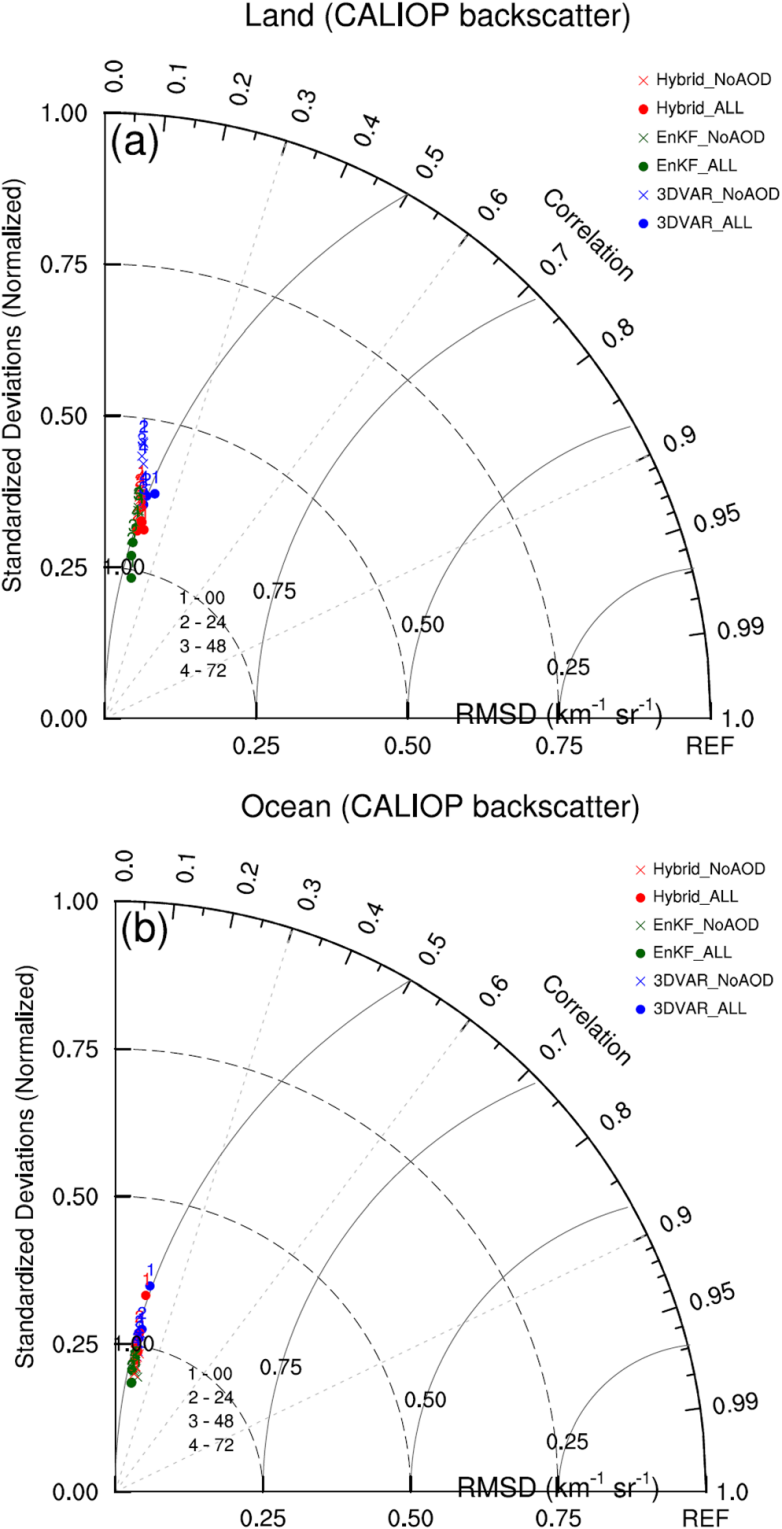
Taylor diagrams comparing simulated backscatter profiles of six DA experiments with CALIOP backscatter observations whose paths cross over (a) land and (b) ocean. Blue, green, and red dots (crosses) denote 3DVAR_ALL, EnKF_ALL, and Hybrid_ALL (3DVAR_NoAOD, EnKF_NoAOD, and Hybrid_NoAOD) experiments, respectively. The numbers 1, 2, 3, and 4 indicate 0‐, 24‐, 48‐, and 72‐hr forecasts, respectively.

Figure 17 shows Taylor diagrams for forecasted backscatter profiles at 0, 24, 48, and 72 hr from all six DA experiments. Taylor statistics for each experiment and forecast time are calculated using vertical profiles of CALIOP backscatter. Over land, the pattern correlation and RMSD values are very similar for all experiments and forecast times with all correlation values between 0.12 and 0.22. Nevertheless, the pattern correlation and RMSD values for the “ALL” experiments are slightly better (i.e., correlation increased; RMSD decreased) than those of the corresponding “NoAOD” experiments at all forecast times. Of the “ALL” experiments, 3DVAR_ALL produces the best backscatter forecasts at 0 and 24 hr, while Hybrid_ALL produces the best forecasts at 48 and 72 hr.

Over the ocean, pattern correlation values are also low for all experiments, falling between 0.14 and 0.20, while normalized standard deviations are slightly worse than those over land, with values between 0.18 and 0.36. RMSDs for all experiments, forecast times, and surface types are very similar.

The conclusions drawn from the Taylor diagrams computed against CALIOP backscatter profiles are noticeably different than those computed against MODIS AOD data for the same forecast times and experiments. MODIS‐generated Taylor diagrams show pattern correlation values between 0.5 and 0.85, substantially higher than those of the CALIOP‐based diagrams, and error (RMSD) values that greatly depend on forecast length. It is possible that the consistent, significant disagreement between modeled and CALIOP backscatter is due not only to inaccurately modeled dust fields but also to potential shortcomings in the G‐SDSU operator, related to the single scattering properties (particularly backscatter) of dust aerosols, used to obtain modeled backscatter from the simulated dust distribution. To better assess the likelihood that the poor Taylor statistics for CALIOP backscatter are due to the G‐SDSU, CALIOP AOD values are compared to those produced by the G‐SDSU.

**Figure 18:**
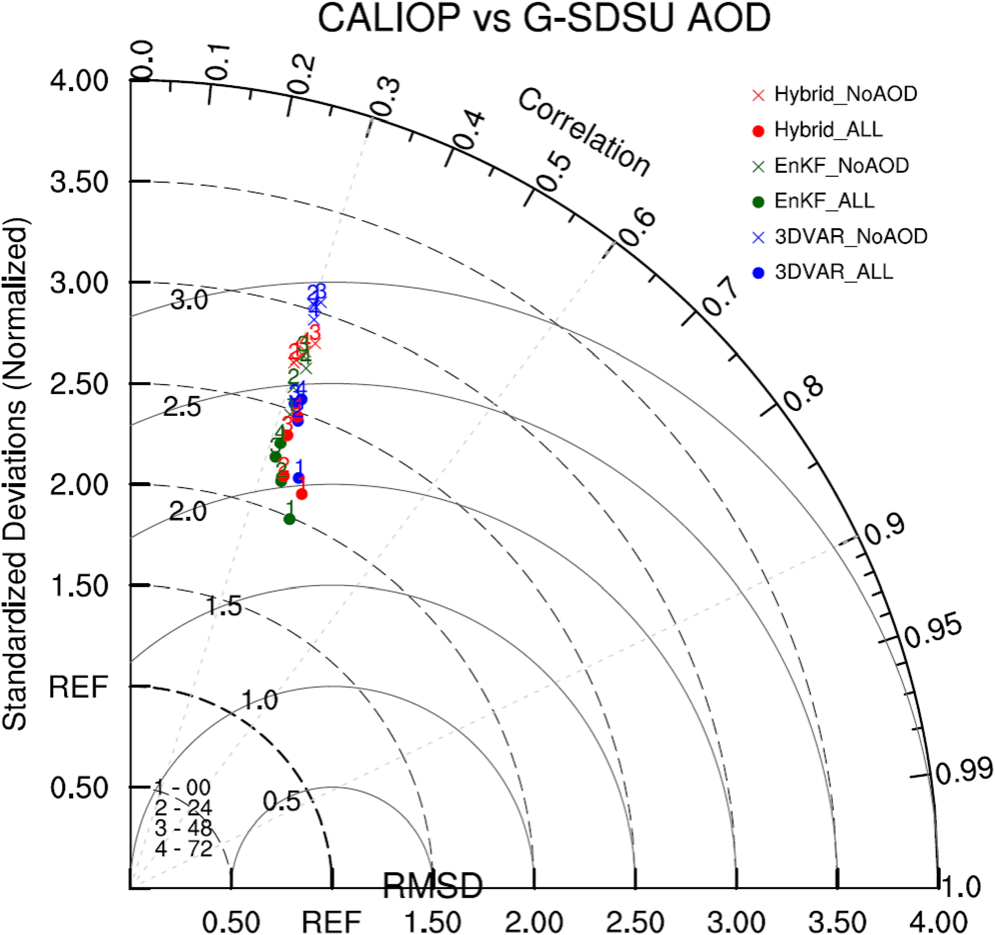
Taylor diagram comparing simulated AOD of six DA experiments with CALIOP AOD observations. Blue, green, and red dots (crosses) denote 3DVAR_ALL, EnKF_ALL, and Hybrid_ALL (3DVAR_NoAOD, EnKF_NoAOD, and Hybrid_NoAOD) experiments, respectively. The numbers 1, 2, 3, and 4 indicate 0‐, 24‐, 48‐, and 72‐hr forecasts, respectively.

Figure 18 shows a Taylor diagram describing the relationship between G‐SDSU‐simulated and CALIOP AOD for all six DA experiments. AOD forecast quality shows clearer dependencies on forecast length and AOD assimilation than the backscatter forecast Taylor statistics. Additionally, AOD assimilation reduces RMSDs for all DA methods and forecast lengths. The fact that MODIS AOD assimilation improves AOD forecasts but has little effect on backscatter forecasts, even though both sets of forecasts are evaluated against CALIOP data and both AOD and backscatter are computed using the G‐SDSU, implies that the consistently poor agreement between forecasted and CALIOP backscatter is largely due to the horizontal, two‐dimensional nature of MODIS AOD observations rather than serious issues in the G‐SDSU backscatter operator. It is this lack of vertical aerosol information that limits the impact of AOD assimilation on backscatter profiles to shifting the entire profile to the left (over land) or right (over ocean).

In short, while MODIS AOD assimilation slightly reduces backscatter RMSEs at all levels for both forecasts and analyses, it reduces the corresponding bias magnitudes at only some levels, with others experiencing an increase in bias magnitudes. The Taylor diagrams show small changes in normalized standard deviations for different forecast times when the 3D‐Var and hybrid methods are used but show no notable variations in correlation or RMSD values between different experiments or forecast lengths. Since MODIS AOD assimilation improves AOD analyses and forecasts with respect to both CALIOP and MODIS observations, its inability to significantly improve backscatter profiles is likely due in part to the fact that MODIS AOD observations do not provide any information regarding the vertical distribution of aerosols.

### 4.3 Impact of Assimilating Deep Blue AOD Data on Analyses and Forecasts

While one would expect deep blue AOD data, via assimilation, to improve dust analyses and forecasts over and downstream of source regions (e.g., desert), deep blue data prior to Collection 6.1 (Sayer et al., 2013, 2014, 2015) were often excluded from assimilation due to the presence of large errors (Levy et al., 2013; Sayer et al., 2013, 2014). Since the release of Collection 6.1, which included deep blue data produced by a significantly improved algorithm, some studies have examined the benefit of assimilating deep blue AOD data over arid regions (e.g., Benedetti et al., 2019; Di Tomaso et al., 2017).

To investigate the impact of deep blue products on dust analyses and forecasts in this study, a seventh experiment is conducted (Hybrid_NoDB) that is identical to Hybrid_ALL but excludes deep blue AOD data (i.e., no AOD data over desert; Figure 1). Results from the Hybrid_NoDB experiment are compared to those from Hybrid_ALL and Hybrid_NoAOD.

Over land, the monthly‐average AOD analysis from Hybrid_NoDB (Figure 4g) is broadly similar to that from Hybrid_NoAOD, though some differences are evident at finer scales with Hybrid_NoDB AOD slightly more consistent with MODIS AOD. Over ocean, the average Hybrid_NoDB AOD analysis is similar to that from Hybrid_ALL except in a small portion of the East Atlantic close to the coast of West Africa where AOD values are larger than in Hybrid_ALL due to greater dust transport from the Sahara Desert, which is the result of higher AOD values over the region caused by the absence of AOD assimilation in Hybrid_NoDB. The Taylor diagram in Figure 12 shows that the analyses and forecasts of Hybrid_NoDB lie between those of Hybrid_ALL and Hybrid_NoAOD, in terms of performance against MODIS AOD, as anticipated. However, the Taylor statistics of Hybrid_NoDB are closer to those of Hybrid_NoAOD, particularly for longer forecast times, demonstrating the importance of assimilating AOD data over major source regions for accurate dust transport forecasts, even when the source region covers a relatively small portion of the total domain.

**Figure 19:**
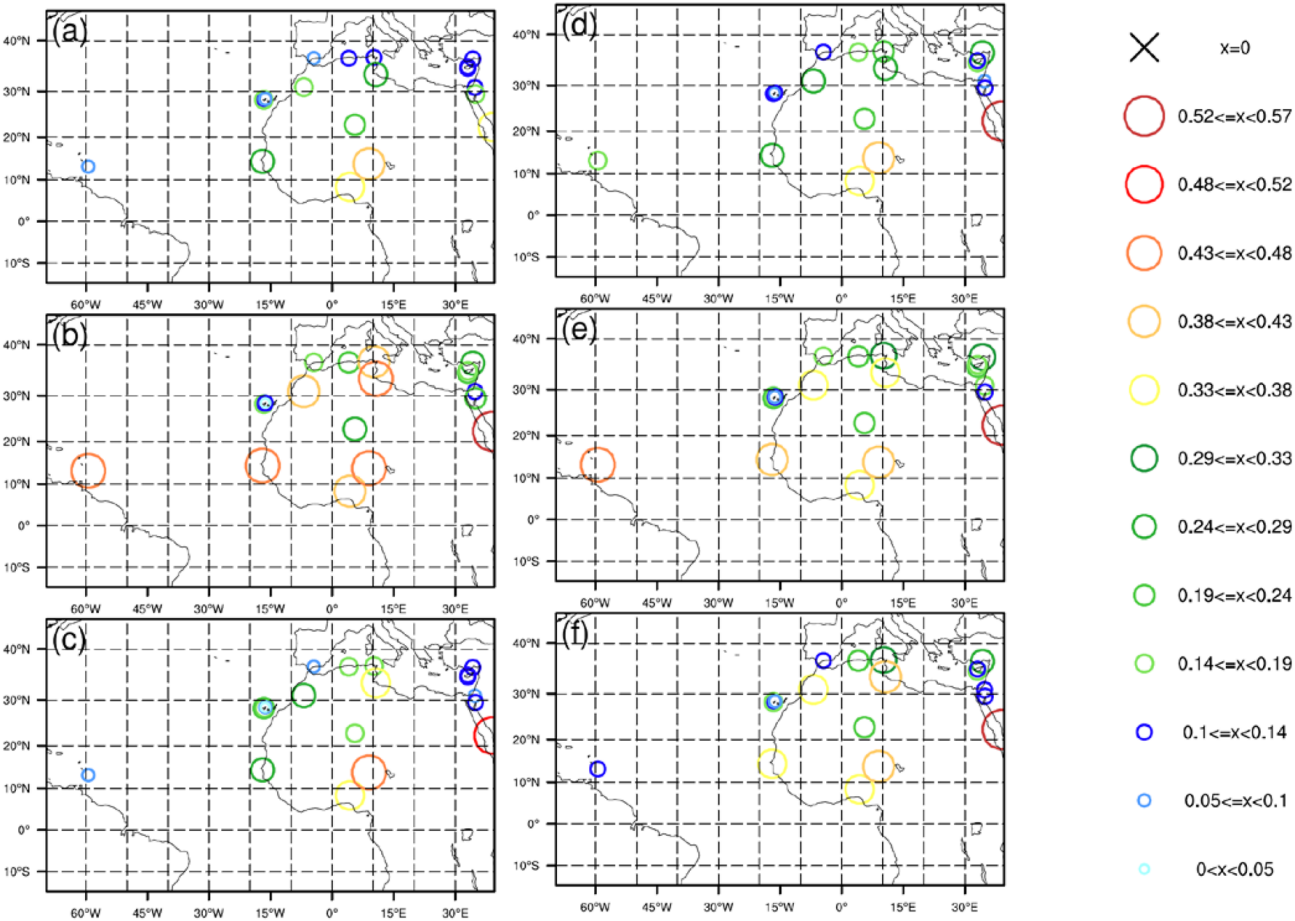
AOD RMSEs for (a–c) analyses and (d–f) 24‐hr forecasts of (a and d) Hybrid_ALL, (b and e) Hybrid_NoAOD, and (c and f) Hybrid_NoDB experiments. RMSEs are calculated against AERONET AOD observations.

Comparing the RMSEs of the three “Hybrid” experiments at individual AERONET sites (Figure 19) reveals that the assimilation of deep blue AOD data reduces analysis and/or forecast errors at many locations. Deep blue AOD assimilation reduces the average 24‐hr forecast RMSEs from the four Canary Island AERONET stations (stations 3, 4, 7, and 10), with four‐station average RMSEs for Hybrid_ALL, Hybrid_NoAOD, and Hybrid_NoDB of 0.097, 0.145, and 0.123, respectively. The RMSE difference between Hybrid_ALL and Hybrid_NoDB is particularly notable since the forecasts of both are derived from analyses that assimilate dark target AOD data over the Canary Islands. That the short‐term forecast errors are still worse for Hybrid_NoDB reinforces the importance of assimilating AOD data over dust source regions (i.e., deep blue AOD data) to ensure accurate dust transport forecasts over both short and long distances. When averaged across all 20 AERONET stations, MAEs and RMSEs are smallest for Hybrid_ALL and greatest for Hybrid_NoAOD, with those of Hybrid_NoDB falling in between (Figure 14), consistent with Taylor statistics computed against MODIS AOD. However, while both Hybrid_ALL and Hybrid_NoDB clearly produce better AOD analyses and forecasts relative to AERONET data, AOD‐assimilation‐driven AOD forecast improvements only persist for 30 hr when deep blue data are excluded, as opposed to 48 hr when both deep blue and dark target data are assimilated (Figure 14b).

## 5 Summary and Conclusions

To assess the impact of assimilating AOD observations with various DA methods over desert areas, we conduct seven numerical experiments using the WRF dust model and the GSI analysis system, each producing analyses and 72‐hr forecasts covering North Africa and the East Atlantic every 6 hr throughout the month of June 2015. Six of the experiments are defined by whether or not AOD observations are assimilated (“ALL” and “NoAOD,” respectively) and the DA method used (3D‐Var [3DVAR], EnSRF [EnKF], and hybrid [Hybrid]). The seventh experiment assimilates AOD data using the hybrid DA method but excludes deep blue data (“NoDB”). Dust analyses and forecasts from all experiments are evaluated against MODIS AOD, AERONET AOD, and CALIOP total backscatter observations. The results of this study are briefly summarized below.

- Comparing the “ALL” experiments to their corresponding “NoAOD” counterparts reveals that assimilating MODIS AOD observations has a positive impact on dust analyses and forecasts, regardless of the DA method used. Specifically, AOD assimilation improves the monthly averaged horizontal AOD distribution, the time evolution of AOD at point locations, and, to a much lesser extent, the profiles of total backscatter relative to MODIS AOD, AERONET AOD, and CALIOP backscatter observations, respectively. AOD assimilation improves AOD forecasts for up to 48 hr, in agreement with Benedetti et al. (2019).
- The relative performance of the analyses and forecasts from the “ALL” experiments against MODIS AOD, AERONET AOD, and CALIOP backscatter data is summarized in Table 5. Hybrid_ALL and EnKF_ALL produce better AOD analyses and forecasts with respect to both MODIS and AERONET AOD observations. On the other hand, 3DVAR_ALL and Hybrid_ALL produce the best analyses and forecasts, respectively, of total backscatter when compared against CALIOP observations. The EnSRF method produces the worst backscatter analyses and forecasts partially due to the fact that it is the only method that lacks an adjoint operator, which, along with the BEC, helps to more precisely distribute the horizontal column AOD increment in the vertical dimension. Overall, the hybrid method performs slightly better than the other two since it benefits from ensemble‐based, flow‐dependent BECs while using static BECs to mitigate sampling problems that can hinder their effectiveness. However, there is no single method that performs best against all three aerosol observation types. It should be noted that the observations used to verify our model results have inherent errors that are not considered in this study but could affect our conclusions.
- Comparing the Hybrid_ALL, Hybrid_NoAOD, and Hybrid_NoDB experiments shows that the assimilation of deep blue AOD data improves AOD analyses and forecasts over most areas near and downstream of dust source regions and causes AOD forecast improvements to persist for an additional 18 hr compared to those where only dark target data are assimilated. This suggests that the usefulness of AOD assimilation can be maximized by using both dark target and deep blue AOD data.

**Table 5:** Summary of Relative Performance of Analyses and Forecasts of “ALL” Experiments With the Top the Best

	MODIS AOD	AERONET AOD	CALIOP backscatter
Analysis	Hybrid_ALL	Hybrid_ALL	3DVAR_ALL
	EnKF_ALL	EnKF_ALL	Hybrid_ALL
	3DVAR_ALL	3DVAR_ALL	EnKF_ALL
24‐hr forecast	EnKF_ALL	Hybrid_ALL	3DVAR_ALL
	Hybrid_ALL	EnKF_ALL	Hybrid_ALL
	3DVAR_ALL	3DVAR_ALL	EnKF_ALL
48‐hr forecast	EnKF_ALL	Hybrid_ALL	Hybrid_ALL
	Hybrid_ALL	EnKF_ALL	3DVAR_ALL
	3DVAR_ALL	3DVAR_ALL	EnKF_ALL
72‐hr forecast	EnKF_ALL	EnKF_ALL	Hybrid_ALL
	Hybrid_ALL	Hybrid_ALL	3DVAR_ALL
	3DVAR_ALL	3DVAR_ALL	EnKF_ALL

This is our first attempt to improve dust forecasts using data assimilation. While some promising and interesting results are obtained, dust analyses and forecasts can be further improved by (1) accounting for aerosols other than mineral dust, which may be especially useful in areas where dust is not the dominant aerosol type, (2) assimilating CALIOP backscatter data to constrain the vertical distribution of aerosols and complement the two‐dimensional, horizontal aerosol information provided by MODIS AOD data, (3) optimizing the EnSRF method by adjusting horizontal/vertical localization scales and implementing more sophisticated inflation methods, and (4) adjusting the wind‐speed threshold for dust emission. All of these issues will be addressed in future work. We will also investigate the effects of assimilating aerosol observations on meteorological forecasts through aerosol‐radiation‐cloud feedbacks.
